# Comparative Transcriptomic Profiling of Diabetic Wound Responses to Recombinant *Periplaneta americana* Thymosin Isoforms in Male ob/ob Mice

**DOI:** 10.3390/genes17101296

**Published:** 2026-10-16

**Authors:** Zhoulong Chen, Lin Yan, Guangqin Zhao, Lei Chen, Yanni Zhao, Shunri Jiang, Chenjuan Zeng, Jingheng Wang, Yushuo Zhang, Bisong Yue, Xiuyue Zhang

**Affiliations:** 1 Key Laboratory of Bio-Resources and Eco-Environment, Ministry of Education, College of Life Sciences, Sichuan University, Chengdu 610064, China; 2 *Periplaneta americana* and Innovative Drugs Key Laboratory of Sichuan Province, Good Doctor Pharmaceutical Group Co., Ltd., Chengdu 610064, China

**Keywords:** diabetic wound, *Periplaneta americana* thymosin, angiogenesis, re-epithelialization, collagen remodeling, transcriptomic

## Abstract

**Background:** Preparations derived from *Periplaneta americana*, such as Kangfuxin Liquid, have been used in China for wound and ulcer management, yet their active constituents remain incompletely characterized. Recombinant *P. americana* thymosin isoforms (Pa-THYs) have been reported to accelerate wound closure in nondiabetic mice. Their effects and associated transcriptional responses in diabetic wounds remain unclear. **Methods:** Three Pa-THYs (THY1–3) were topically applied to full-thickness excisional wounds in ob/ob diabetic mice, with thymosin β4 (Tβ4) and PBS used as positive and negative controls. Wound healing was assessed by planimetry, hematoxylin and eosin (H&E) staining, Masson trichrome staining, and CD31 immunohistochemistry. RNA sequencing of wound tissues on days 3 and 8 post-injury was performed, followed by differential gene expression analysis, Gene Ontology and pathway enrichment analysis, protein–protein interaction network construction, marker gene-based cell-type-associated expression scoring, and preranked GSEA. **Results:** On day 8, mean wound-closure percentages ranged from 95% to 97% across the THY1, THY2, THY3, and Tβ4 groups compared to 81% in the PBS group. At this time point, treated wounds showed more complete epithelial coverage. The CD31-positive area fraction was higher in all active-treatment groups, whereas the collagen volume fraction was higher in the THY2 and THY3 groups than in the PBS group. Among the Pa-THY groups, THY2 showed the largest day-3 DEG set, whereas THY1 and THY3 showed prominent epithelial-differentiation signatures at the later time point. THY3 showed the largest day-8 DEG set and the greatest pairwise DEG overlap with Tβ4. As a complementary threshold-independent analysis, GSEA identified coordinated early metabolic responses, broadly distributed interferon-associated enrichment, and late keratinization enrichment in THY1, THY3, and Tβ4. **Conclusions:** Topical THY1, THY2, and THY3 improved day-8 wound closure in male ob/ob mice, with accompanying changes in epithelial coverage, CD31-positive area fractions, and collagen staining. The transcriptomic findings indicate shared pathway-level responses together with time- and isoform-associated differences. These results support a further preclinical evaluation of Pa-THYs.

## 1. Introduction

Diabetes mellitus is a major risk factor for chronic, non-healing wounds. Approximately 20–30% of diabetic patients will develop a chronic cutaneous ulcer (such as a diabetic foot ulcer, DFU) in their lifetime [1,2]. These chronic wounds impose a tremendous clinical and economic burden, with high recurrence rates, prolonged wound care, and costly hospitalizations [3]. Normal wound healing requires coordinated processes, such as angiogenesis, immune regulation, and re-epithelialization [4]. However, these processes are impaired in diabetic wounds. Diabetes-associated vascular dysfunction can impair angiogenesis and reduce blood flow and oxygen delivery to the wound site. Immune dysregulation in diabetic patients leads to a long-term inflammatory response and the wound usually remains in a chronic inflammatory state [5]. Moreover, hyperglycemic conditions can impair keratinocyte and fibroblast function, which are essential for epithelial regeneration, resulting in delayed wound surface closure [6,7]. Diabetic wounds also exhibit reduced infection control, and a hyperglycemic environment favors sustained bacterial colonization, making these wounds prone to non-resolving infections [8]. Collectively, impaired angiogenesis, persistent inflammation, and defective epithelial regeneration contribute to delayed wound repair.

At present, there are many methods for the treatment of chronic diabetic wounds, including wound dressings, small-molecule drugs, and skin grafts. Despite advances in wound care, few agents have been found to substantially improve chronic wound healing outcomes in diabetic patients [9]. Topical growth factors and plant-derived small-molecule drugs are among the treatments currently being explored [10]. However, these approaches have limitations: growth-factor therapies can be costly and may have limited stability, whereas the in vivo activity of small-molecule compounds varies between agents. One candidate that has been investigated for wound repair is thymosin β4 (Tβ4), a naturally occurring 43-amino-acid peptide with pleiotropic regenerative activities [11]. Tβ4 is known to promote wound repair, angiogenesis, and tissue regeneration, while also exerting anti-inflammatory effects [12]. These reported angiogenic and regenerative activities have prompted investigation of Tβ4 as a potential treatment for chronic and difficult-to-heal wounds.

*P. americana* (the American cockroach) has attracted interest as a source of bioactive molecules. Studies of *P. americana* extracts have reported antifibrotic and wound-repair-associated activities [13,14]. *P. americana*-derived preparations, including Kangfuxin Liquid, have been used in China for wound and ulcer management [15,16]. However, most *P. americana*-based medicines currently on the market are crude extracts with complex compositions. Their active constituents have not been fully defined, which limits quality control, batch reproducibility, and mechanistic interpretation [17]. Identifying defined bioactive components may improve their characterization and help distinguish the contributions of individual molecules to tissue repair. Recombinant Pa-THYs provide an opportunity to examine defined isoforms separately from the broader activities of *P. americana* extracts.

Thymosin isoforms derived from *P. americana* (Pa-THYs) are structurally homologous to mammalian Tβ4. Jing et al. reported that topical application of three recombinant Pa-THY isoforms (THY1, THY2 and THY3) accelerated full-thickness skin wound closure in nondiabetic mice (*Mus musculus*), with dermal regeneration, angiogenesis, and collagen deposition comparable to Tβ4 in the same model [18]. Whether these isoforms retain their wound-repair activity in diabetic wounds and whether their associated transcriptional responses differ remain unclear. This study aimed to compare the wound-repair outcomes and treatment-associated transcriptional responses of THY1, THY2, and THY3 in male ob/ob mice, using PBS and Tβ4 as controls. We examined shared and isoform-associated expression patterns to characterize responses at earlier and later stages of diabetic wound repair.

## 2. Methods

### 2.1. Experimental Animals and Feeding

Male SPF ob/ob diabetic mice, weighing 36–40 g and aged 7–8 weeks, were obtained from Beijing HuaFukang Biotechnology Co., Ltd. (Beijing, China). The experimental animals were produced under license number SCXK (Beijing) 2019-0008. SPF-grade feed and bedding materials were obtained from Chengdu Dashuo Experimental Animal Co., Ltd. (Chengdu, China). The mice were housed in chambers maintained at a temperature of 20–24 °C and a relative humidity of 30–50%. The bedding materials were regularly changed, and a 12 h light/dark cycle was upheld. Mice were housed two per cage, with unrestricted access to SPF-grade feed and drinking water. Seventy-five male ob/ob mice were assigned to five groups, with 15 mice per group. Three mice per group were randomly selected for tissue collection on days 3 and 5. Wound-closure analysis included nine mice per group that had completed follow-up through to day 8, of which three were selected for tissue collection on day 8. No unplanned deaths or replacement animals occurred. All study procedures and animal care activities were approved by the Academic and Ethics Committee of Sichuan University (license number SCU230109001; approved on 5 June 2020).

### 2.2. Recombinant Protein Production and Purification

Three recombinant Pa-THY isoforms were produced in *E. coli* using previously described expression and purification procedures. The sequence-predicted molecular masses of Pa-THY1–3 were approximately 19.0, 14.6, and 23.4 kDa [18]. The Pa-THY coding sequences were cloned into pET-28a(+), and the resulting expression plasmids were transformed into *E. coli* BL21 cells. The recombinant strains were stored as glycerol stocks at −80 °C. For expression, the strains were cultured in kanamycin-supplemented LB at 37 °C with shaking. Overnight cultures were diluted 1:100 into fresh medium and grown to an OD_600_ of 0.5–0.8, followed by IPTG induction for 6 h at 37 °C. The cells were harvested via centrifugation, resuspended in 40–50 mL of PBS, lysed via sonication, and clarified via centrifugation. The supernatant was passed through a 0.22 μm filter and purified via a HisTrap^TM^ FF crude column. After washing with 30 mM imidazole for 20 min, the proteins were eluted with 300 mM imidazole. The eluates were concentrated by ultrafiltration (4000 r/min), and the buffer was changed to PBS by repeated dilution/concentration cycles to remove imidazole. Protein preparations were examined by SDS–PAGE with Coomassie blue staining, and protein concentrations were determined using a BCA assay. The preparations were aliquoted and stored at −80 °C until use.

### 2.3. Establishment of the Diabetic Mouse Wound Model

Male ob/ob mice were acclimatized for 1 week. After a 12 h fast, blood was collected from the tail vein to measure fasting blood glucose. Mice with fasting blood glucose values exceeding 11.1 mmol/L were classified as diabetic models [19,20]. A total of 75 diabetic mice were randomly assigned to five groups (15 mice per group): a negative control (PBS control group), three treatment groups (THY1, THY2 and THY3), and a positive control (Tβ4-treated group). Surgical instruments were sterilized before use. After the mouse was anesthetized, the dorsal fur was trimmed, and depilatory cream was applied for 1–2 min. Residual hair and cream were then removed with a cotton ball, and the exposed skin was disinfected. A single circular full-thickness wound (approximately 1.2 cm in diameter, extending to the fascial layer) was created on the back of each mouse, centered on the dorsal midline. Beginning 24 h after wound creation, THY1, THY2, THY3, or Tβ4 was applied topically once daily at a nominal dose of 25 μg/cm^2^ [18]. PBS was applied to control wounds. Wounds were photographed every 2 days (days 0, 2, 4, 6, and 8 after wounding). Day 0 was the day of wound creation; all subsequent study days were counted from this day.

### 2.4. Calculation of Wound-Closure Percentage and Statistical Analysis

Dorsal wound areas were measured using Image-Pro Plus 6.0 on days 0, 2, 4, 6, and 8 after wounding. Each area was measured three times, and the measurements were averaged. Wound-closure analyses included the same nine mice per group with complete 8-day follow-up. The mouse was the experimental unit, with one wound per mouse. Data are expressed as mean ± SD. Between-group differences on days 2, 4, 6, and 8 were assessed using two-sided Welch’s t-tests based on the group means and SDs reported in Table S1. Bonferroni correction was applied to the ten pairwise comparisons at each time point, with adjusted *p* < 0.05 considered statistically significant. Mean differences were expressed in percentage points and accompanied by unadjusted 95% confidence intervals (CIs) and Bonferroni-adjusted simultaneous 95% CIs for the comparisons within each time point. Cohen’s d and Hedges’ g were calculated from pooled SDs as descriptive measures of standardized effect size (Table S2). A sensitivity analysis applied Bonferroni correction across all 40 comparisons. Day-0 measurements were excluded from hypothesis testing. Statistical analyses were performed in R version 4.6.1. Wound closure (%) was calculated using the following equation:

$$y(n)=\frac{A_{0}-A_{n}}{A_{0}}\times 100\%$$

where *y* is the healing rate on day *n* (%); *A*_0_ is the initial wound area (cm^2^); and *A_n_* is the wound area on day *n* (cm^2^).

### 2.5. Sample Collection

Wound tissues were collected on days 3, 5, and 8 after wound creation following euthanasia by cervical dislocation (*n* = 3 mice per group per time point). The day-8 samples were obtained from three of the nine mice per group that completed the 8-day wound-closure follow-up. Tissues encompassing the wound and a 1–2 mm margin of adjacent normal skin were excised using sterilized scissors and divided into two portions. One portion was snap-frozen in liquid nitrogen for RNA extraction, whereas the other was fixed in 4% paraformaldehyde for histological analysis.

### 2.6. Histopathology and Immunohistochemistry Experiments

Skin tissues fixed in 4% paraformaldehyde for 24 h were submitted to Aochuang (Chengdu) Biological Company (Chengdu, China) for hematoxylin and eosin (H&E) staining, CD31 immunohistochemical analysis, and Masson staining. CD31 immunostaining was quantified as the percentage of CD31-positive area relative to the total tissue area. Masson staining analysis was conducted by calculating the collagen volume fraction (CVF), defined as the proportion of blue-stained collagen area to the total tissue area [21]. The positive and total tissue areas for both CD31 immunohistochemical and Masson staining were quantified using ImageJ software (version 1.52). Five fields of view were analyzed per sample, and their values were averaged to obtain one sample-level measurement. The mouse was the experimental unit (*n* = 3 mice per group per time point). CD31-positive area fractions and CVFs were analyzed by one-way ANOVA at each time point using GraphPad Prism 8.4.0, with *p* < 0.05 considered to be significant.

### 2.7. Total RNA Extraction and RNA Library Preparation

Days 3 and 8 after wounding were selected before the study for RNA-seq to examine early and late wound responses during the 8-day observation period. Total RNA was extracted from wound tissue, including a 1–2 mm margin of adjacent normal skin, using the RNAPREP Pure tissue kit (Tiangen, Beijing, China). Each of the five groups contributed three samples from separate mice at each sequencing time point (*n* = 3), yielding 30 biological samples without pooling. All samples were processed in a single batch at each stage of RNA extraction, library preparation, and sequencing. RNA integrity was initially assessed by agarose gel electrophoresis of samples from 10 randomly selected ob/ob mice, which showed bands corresponding to 28S, 18S, and 5S ribosomal RNA (Figure S4). Samples were submitted to Novogene (Beijing, China) for further quality assessment, library preparation, and sequencing on the Illumina NovaSeq 6000 platform. Raw sequencing data are available in CNGBdb under accession CNP0003984 (https://db.cngb.org/data_resources/project/CNP0003984) (accessed on 4 February 2023). Sample-level sequencing QC metrics from the archived FastQC (version 0.12.1) reports, as summarized in MultiQC (version 0.4), are provided in Table S3.

### 2.8. Raw Sequencing Data Quality Control and Gene Expression Quantification

Raw data underwent quality control using the NGS QC Toolkit (version 2.3.3), which eliminated low-quality and short reads [22]. HISAT2 (version 2.2.1) software was used to map the reads to the reference genome [23]. The mouse reference genome (GRCm39) and its annotations were retrieved from Ensembl Release 115 (https://ftp.ensembl.org/pub/release-115/) (accessed on 18 February 2026). SAMtools (version 1.21) was employed to process the alignment files into BAM format for further analyses [24]. The featureCounts tool from the Subread package (version 2.0.6) was used to quantify the gene expression levels based on the mouse reference genome, preparing the data for subsequent analysis [25].

### 2.9. Principal Component Analysis and Hierarchical Clustering

PCA was used to examine overall expression patterns across treatments and time points. Raw read counts were processed with edgeR (version 4.6.3). Lowly expressed genes were filtered using filterByExpr, and counts were normalized by the trimmed mean of M values (TMM) method and converted to log_2_ counts per million (log_2_-CPM). PCA was performed on the transposed expression matrix using prcomp, with genes centered and scaled. The first two components were plotted with ggplot2 (version 4.0.0), and axis labels indicate the proportion of variance explained.

For hierarchical clustering analysis, pairwise Spearman correlation coefficients were calculated from the log_2_-CPM matrix using cor in the stats package. A heatmap with hierarchical clustering was generated using the heatmap.2 function from the gplots (version 3.3.0) package.

### 2.10. Differential Gene Expression Analysis

To identify changes in gene expression in the wound skin of mice treated with thymosin from *P. americana*, transcriptome data from THYs, PBS, and Tβ4-treated groups on days 3 and 8 after wounding were analyzed in this study. Eight treatment-versus-time-matched PBS contrasts were analyzed: THY1, THY2, THY3, and Tβ4 versus PBS on days 3 and 8. The R package stats (v4.5.2) was used for PCA and hierarchical clustering analysis. The differential expression analysis was performed using DESeq2 (version 1.48.1) [26]. Genes with a Benjamini–Hochberg-adjusted *p* value < 0.05 and an absolute log_2_ fold change > 1 were classified as DEGs. To visualize the expression patterns of differentially expressed genes, we performed hierarchical clustering heatmap analysis using day-3 and day-8 samples. First, the gene expression data were Z-score standardized to eliminate the difference in expression magnitude between different genes, and the genes were clustered by unsupervised hierarchical clustering. The heatmaps were generated using the default parameters of the pheatmap package (version 1.0.13). Mean–dispersion relationships were examined in models reconstructed for the eight original treatment-versus-time-matched PBS contrasts (Figure S18).

### 2.11. Functional Enrichment Analysis

GO and KEGG over-representation analyses were conducted separately for upregulated and downregulated DEG sets using g:Profiler (https://biit.cs.ut.ee/gprofiler/) (accessed on 20 February 2026). Benjamini–Hochberg-adjusted *p* values < 0.05 were considered significant. Preranked GSEA was conducted separately for each treatment-versus-time-matched PBS comparison using clusterProfiler (version 4.20.0) with the multi-level algorithm implemented in enrichit (version 0.2.0). Genes were ranked according to the DESeq2 Wald statistic, and gene sets containing 15–500 genes were evaluated using a weighted enrichment statistic and an exponent of 1. Using the Benjamini–Hochberg method, *p* values were adjusted within each contrast and gene-set collection, with an FDR below 0.05 considered significant. Analyses were performed independently for days 3 and 8; no formal treatment–time interaction was tested.

### 2.12. Protein Interaction Network Analysis

To further examine interactions among proteins encoded by the DEGs, we used the STRING database (https://cn.string-db.org/) (accessed on 21 February 2026). Network analysis and hub gene screening were conducted using Cytoscape (v3.10.4) [27]. Protein interaction data obtained from the STRING database were filtered using default parameters (interaction score > 0.4), and interaction relationship files were exported. Cytoscape software was used to construct PPI network diagrams for DEGs. Further network analysis tools in Cytoscape were employed to calculate the connectivity (degree) of each gene node. Genes with relatively high degree centrality were described as network hub genes.

### 2.13. Cell-Type-Associated Marker Expression Scoring

Cell-type-associated marker expression scores were calculated from bulk RNA-seq data using a custom marker gene-based approach inspired by the mMCP-counter framework [28] and xCell [29]. Custom mouse marker gene sets were used for 12 cell populations: T cells, CD8 T cells, NK cells, B cells, monocytes/macrophages, monocytes, macrophages, neutrophils, eosinophils, mast cells, fibroblasts, and endothelial cells. The marker gene sets and the genes detected in this dataset are listed in Table S17. Raw counts were filtered to remove all-zero genes, deduplicated by retaining the row with the highest mean expression for each gene symbol, and normalized to CPM. For each sample and cell type, the score was computed as the mean of log_2_(CPM + 1) across detected markers, requiring at least two detected markers per cell type. Scores are comparable between samples within a given cell type. Within each time point and cell type, each treatment group was compared with the PBS control using a two-sided exact Wilcoxon rank-sum test, with Benjamini–Hochberg adjustment applied for each (time point × cell type) stratum. Effect sizes were reported as Hedges’ g, and comparisons with |g| > 1.0 were highlighted as exploratory effect-size signals.

## 3. Results

### 3.1. Macroscopic and Histological Assessment of Wound Healing

**Figure 1:**
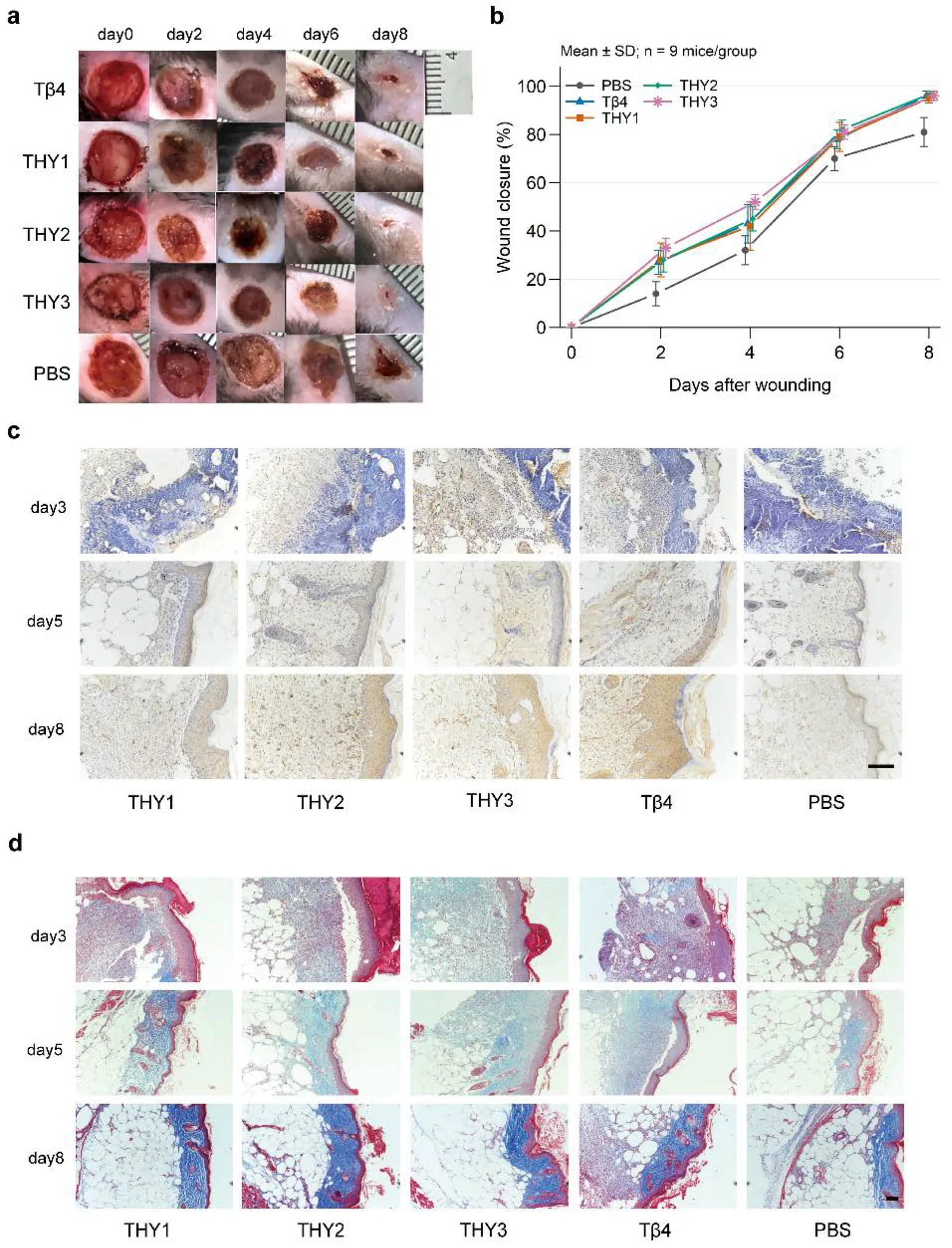
Wound closure and histology. (**a**) Wound photographs. (**b**) Closure (mean ± SD; *n* = 9 mice/group; one wound/mouse); within-day Bonferroni-adjusted Welch’s tests (Table S2). (**c**,**d**) Representative CD31 and Masson staining on days 3, 5, and 8 (×200/×100; scale bars, 50/100 μm). Quantification: Figures S1 and S2 (*n* = 3 mice/group/time point; five fields averaged/mouse). Days are counted from wounding.

Wounds treated with Pa-THY isoforms or Tβ4 appeared smaller and showed earlier scab formation than PBS-treated wounds (Figure 1a). Mean wound closure was higher in all four treatment groups than in PBS at each follow-up assessment (Figure 1b; Table S1). After Bonferroni correction, all treatment–PBS comparisons were significant except THY1 on day 4. THY3 had the highest mean closure on days 2 and 4. On day 4, its mean was significantly higher than that of THY2 by seven percentage points, but did not differ significantly from THY1 or Tβ4. By day 8, wounds in all four treatment groups were nearly closed, with mean closure ranging from 95% to 97% across groups. In contrast, PBS-treated wounds remained partially open with adherent scabs, and mean closure was 81%. No significant differences among the four treatments were detected at this time point, whereas their differences from PBS remained significant in the sensitivity analysis. Pairwise comparisons and confidence intervals are reported in Table S2.

Histological evaluation was performed to characterize the wound healing process. Representative H&E-stained sections showed necrotic tissue and inflammatory-cell-rich areas in Pa-THY- and Tβ4-treated wounds on day 3 (Figure S3). By day 8, Pa-THY- and Tβ4-treated wounds showed more complete epithelial coverage than PBS-treated wounds, which retained incomplete coverage and widened epidermal–dermal gaps. Hair follicle structures were visible in the sampled sections. CD31-positive area fractions were significantly higher in the THY2, THY3, and Tβ4 groups than in PBS on day 5 (Figure 1c and Figure S1). On day 8, all Pa-THY- and Tβ4-treated groups showed significantly higher CD31-positive area fractions than PBS. Masson trichrome staining showed significantly higher collagen volume fractions (CVFs) in the THY2 and THY3 groups than in PBS on day 8, with the highest mean CVF observed in THY2 (Figure 1d and Figure S2). Together, these findings showed greater day-8 wound closure and higher CD31-positive area fractions in the Pa-THY groups than in PBS, with higher CVF in THY2 and THY3. Wound-closure and histological improvements were also reported by Jing et al. in a nondiabetic mouse model [18].

### 3.2. Principal Component Analysis and Spearman Correlation Distance Clustering

**Figure 2:**
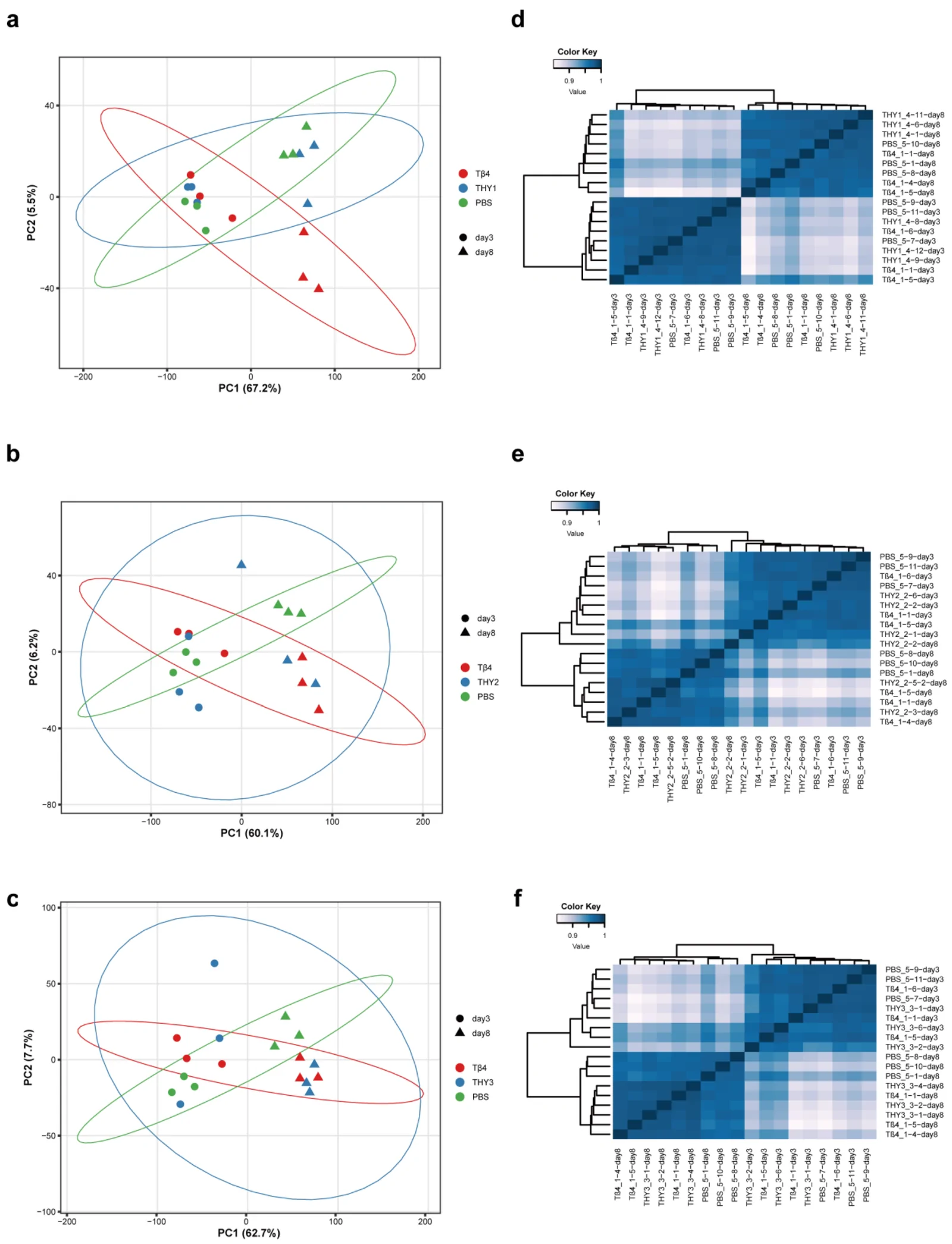
Principal component analysis and sample-to-sample correlations of wound transcriptomes. (**a**–**c**) PCA of THY1, THY2, and THY3 samples, together with the corresponding PBS and Tβ4 controls. Colors indicate treatment groups; circles and triangles indicate days 3 and 8, respectively. Axis labels show the variance explained by each principal component. (**d**–**f**) Corresponding heatmaps of pairwise Spearman correlation coefficients. Each point or heatmap row represents one mouse (*n* = 3 mice per group per time point).

PCA revealed clear separation between treatment groups across all three panels. PC1 explained the majority of variance (60.1–67.2%), while PC2 accounted for 5.5–7.7% of variance (Figure 2a–c). In all three groups, Tβ4-treated samples clustered separately from PBS controls along PC1. THY-treated samples showed intermediate positioning between Tβ4 and PBS groups. Samples were also segregated by time point, with those from days 3 and 8 forming distinct clusters, indicating temporal changes in gene expression. Unsupervised hierarchical clustering confirmed the PCA results. Samples clustered primarily by treatment condition, with high correlation within treatment groups. Day-8 samples separated from day-3 samples across the three analyses, consistent with time-dependent transcriptomic changes. PBS controls maintained stable expression patterns at both time points (Figure 2d–f). PCA and hierarchical clustering showed treatment- and time-associated variation in overall expression profiles. Pa-THY samples showed some overlap with the Tβ4 expression profiles, with patterns varying between treatment groups.

### 3.3. Analysis of Differentially Expressed Genes (DEGs)

**Figure 3:**
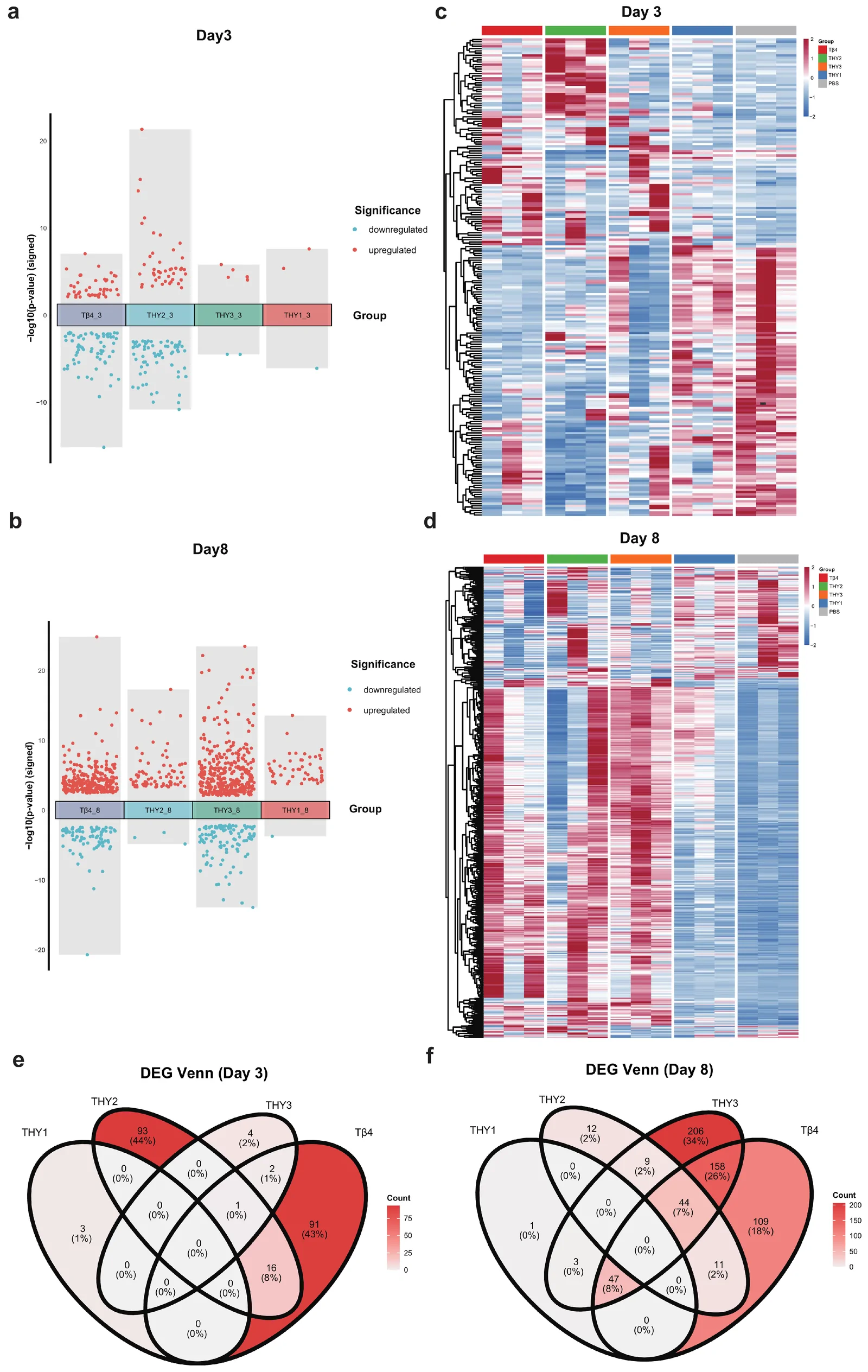
Differential expression patterns after Pa-THY treatment. (**a**,**b**) Each dot represents a DEG. The y−axis shows the signed −log_10_(*p* value), with positive values indicating upregulated genes and negative values indicating downregulated genes. Upregulated genes are colored red, and downregulated genes are colored blue. (**c**,**d**) Heatmaps showing hierarchical clustering of DEGs across samples, with the color scale representing row-wise Z-scaled normalized gene expression (red—high expression, blue—low expression; *n* = 3 per group per time point). (**e**,**f**) Venn diagrams of DEGs across treatment groups on days 3 and 8; color intensity reflects the number of DEGs in each region.

To characterize the transcriptional responses elicited by Pa-THYs and Tβ4 in diabetic wound tissue, DEGs were identified between each treatment group and the PBS control on days 3 and 8 post-wounding and visualized using signed significance plots and heatmaps (Figure 3 and Figure S5). The number of DEGs differed between treatment groups and time points (Table S4). The THY1 and THY3 comparisons contained three and seven DEGs on day 3, respectively, compared with 51 and 467 (predominantly upregulated) on day 8. Tβ4 showed a similar trend (110 on day 3; 369 on day 8). In contrast, THY2 produced 110 DEGs on day 3 and declined to 76 on day 8. Venn diagram analysis showed contrasting overlap patterns at the two time points (Figure 3). On day 3, most DEGs were detected in only one treatment-versus-PBS contrast under the specified DEG criteria. THY2 and Tβ4 accounted for 93 (44%) and 91 (43%) of all DEGs, respectively, with negligible overlap between any pair of groups. By day 8, intergroup overlap increased considerably. THY3 contributed the largest set of unique DEGs (206, 34%) and shared 158 DEGs (26%) with Tβ4, constituting the largest pairwise overlap at either time point. Both three-way DEG intersections on day 8 included THY3 and Tβ4. One comprised 47 DEGs (8%) shared with THY1 while the other comprised 44 DEGs (7%) shared with THY2. No DEGs were shared exclusively among THY1, THY2, and THY3 under the specified DEG criteria. Cell-type-associated marker expression scores were calculated from the same RNA-seq data to characterize myeloid-, lymphoid-, and stromal-associated expression patterns. Exploratory between-group differences (*n* = 3) were summarized using Hedges’ g, with |g| > 1.0 highlighted as an effect-size signal. On day 3, THY1 showed higher expression scores for several myeloid-associated marker sets (monocytes/macrophages g = 2.05, monocytes g = 2.46, eosinophils g = 2.36, mast cells g = 1.29), whereas neutrophil-associated scores were lower in the THY3 and Tβ4 groups than in PBS (g = −1.02 and −1.53). By day 8, higher lymphoid-associated marker expression scores were observed in several treatment groups relative to PBS: NK-cell-associated scores were higher in THY2 (g = 1.77) and THY3 (g = 3.32), and T-cell-associated scores were higher in THY3 (g = 1.72), Tβ4 (g = 1.26) and THY2 (g = 1.15). The THY3 group also showed lower macrophage- (g = −1.27), fibroblast- (g = −1.30), and endothelial-cell-associated (g = −1.24) scores than PBS (Figures S7–S10, Table S16). Differences in myeloid- and lymphoid-associated marker expression scores were observed across treatments and time points, with several day-8 contrasts showing higher lymphoid-associated scores.

### 3.4. GO and KEGG Functional Enrichment Analysis

**Figure 4:**
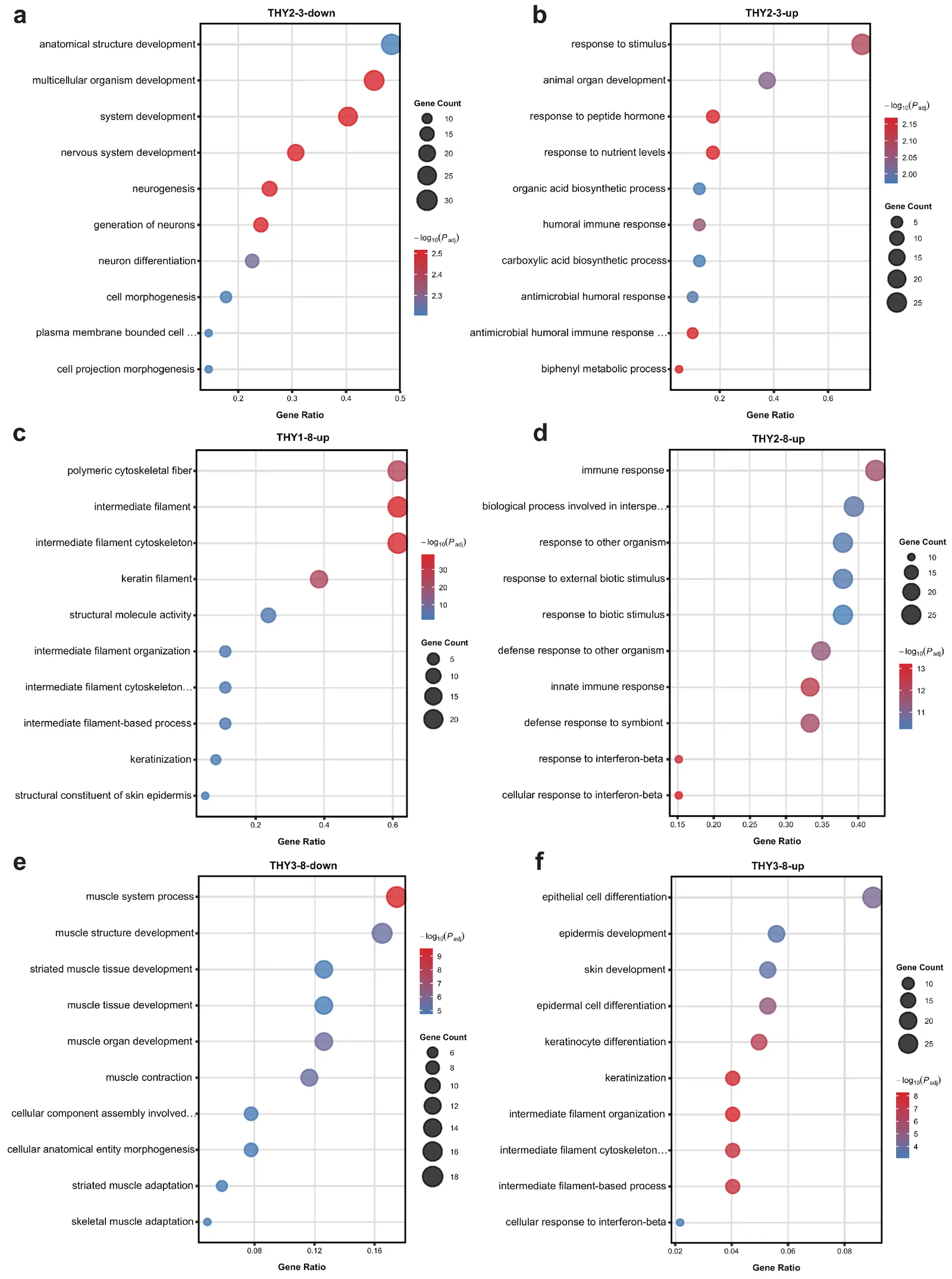
GO enrichment of treatment-associated DEGs. (**a**,**b**) Downregulated and upregulated DEGs in THY2 on day 3. (**c**,**d**) Upregulated DEGs in THY1 and THY2 on day 8. (**e**,**f**) Downregulated and upregulated DEGs in THY3 on day 8. All comparisons used time-matched PBS controls. The dots’ positions indicate the gene ratio, their sizes indicate the DEG count, and their colors indicate −log_10_(adjusted P). RNA-seq: *n* = 3 mice per group per time point.

GO enrichment analysis was performed to characterize the biological processes represented in the treatment-versus-PBS DEG sets. Immune-response-related GO terms, including innate immune response and wound-healing-related processes, were enriched among upregulated DEGs at the sampled time points. On day 3, during early wound repair [30], genes upregulated in the treatment groups were predominantly enriched in cell-proliferation and immune-response pathways. These pathways mainly involved the innate immune response, T-cell and leukocyte activity, and other biological processes. Innate immune response and cytokine pathways were significantly enriched in the THY2-treated group, including the humoral immune response, response to cytokine, antimicrobial humoral response, response to fibroblast growth factor and lymphocyte chemotaxis. Downregulated DEGs were enriched in nervous-system-development and neuron-morphogenesis terms (Figure 4a,b).

By day 8, THY3 and Tβ4 upregulated genes were enriched in a broad range of epidermal and skin development pathways, including keratinization, keratinocyte differentiation, epidermis development, epithelial cell differentiation, and skin development (Figure 4f and Figure S6d). Upregulated DEGs in THY1 and THY3 were enriched in intermediate filament cytoskeleton organization. THY2 did not show significant enrichment of epidermal-development terms on day 8. Instead, its upregulated DEGs were enriched in immune-response, infection-annotated, and interferon-related gene sets. THY3 and Tβ4 were also significantly enriched in interferon-related pathways (cellular response to interferon-beta), showing that interferon-associated enrichment on day 8 was shared by THY2, THY3, and Tβ4. THY3 and Tβ4 showed enrichment of both interferon-response and epidermal-development terms, whereas THY2 showed predominantly immune-related enrichment. Downregulated genes in THY3 and Tβ4 were enriched in muscle contraction, muscle tissue development, and muscle cell differentiation (Figure 4e and Figure S6c), indicating lower relative expression of genes annotated to muscle-related processes in these contrasts. Overall, the day-3 contrasts showed enrichment of immune- and inflammatory-response terms, whereas several day-8 contrasts also showed enrichment of epithelial-development terms. The combinations of enriched terms varied across treatment-versus-PBS comparisons.

KEGG pathway analysis was performed alongside GO enrichment (Tables S5–S15). On day 3, no KEGG pathways were enriched in any Pa-THY-treated group. Only the Tβ4-treated group yielded significant KEGG terms at this time point: upregulated genes were enriched in antigen processing and presentation, cell adhesion molecules, and chemokine signaling pathway, among others, while downregulated genes were enriched in cytokine–cytokine receptor interaction and JAK-STAT signaling pathway. On day 8, KEGG enrichment was detected across multiple treatment groups. Upregulated genes in THY1, THY3, and Tβ4 were commonly enriched in *Staphylococcus aureus* infection and estrogen signaling pathway. THY2 and Tβ4 shared enrichment in the NOD-like receptor signaling pathway and several pathways related to viral infection, including measles, influenza A, and coronavirus disease. In addition, THY2 upregulated genes were uniquely enriched in Epstein–Barr virus infection, hepatitis C, and herpes simplex virus 1 infection. Downregulated genes in THY3 were enriched in calcium signaling pathway and cardiac muscle contraction. Overall, KEGG analysis on day 8 corroborated the GO findings: upregulated DEGs were enriched in several infection-annotated and innate-immune signaling pathways, whereas downregulated DEGs in THY3 were enriched in muscle-related pathways.

### 3.5. Preranked Gene Set Enrichment Analysis

**Figure 5:**
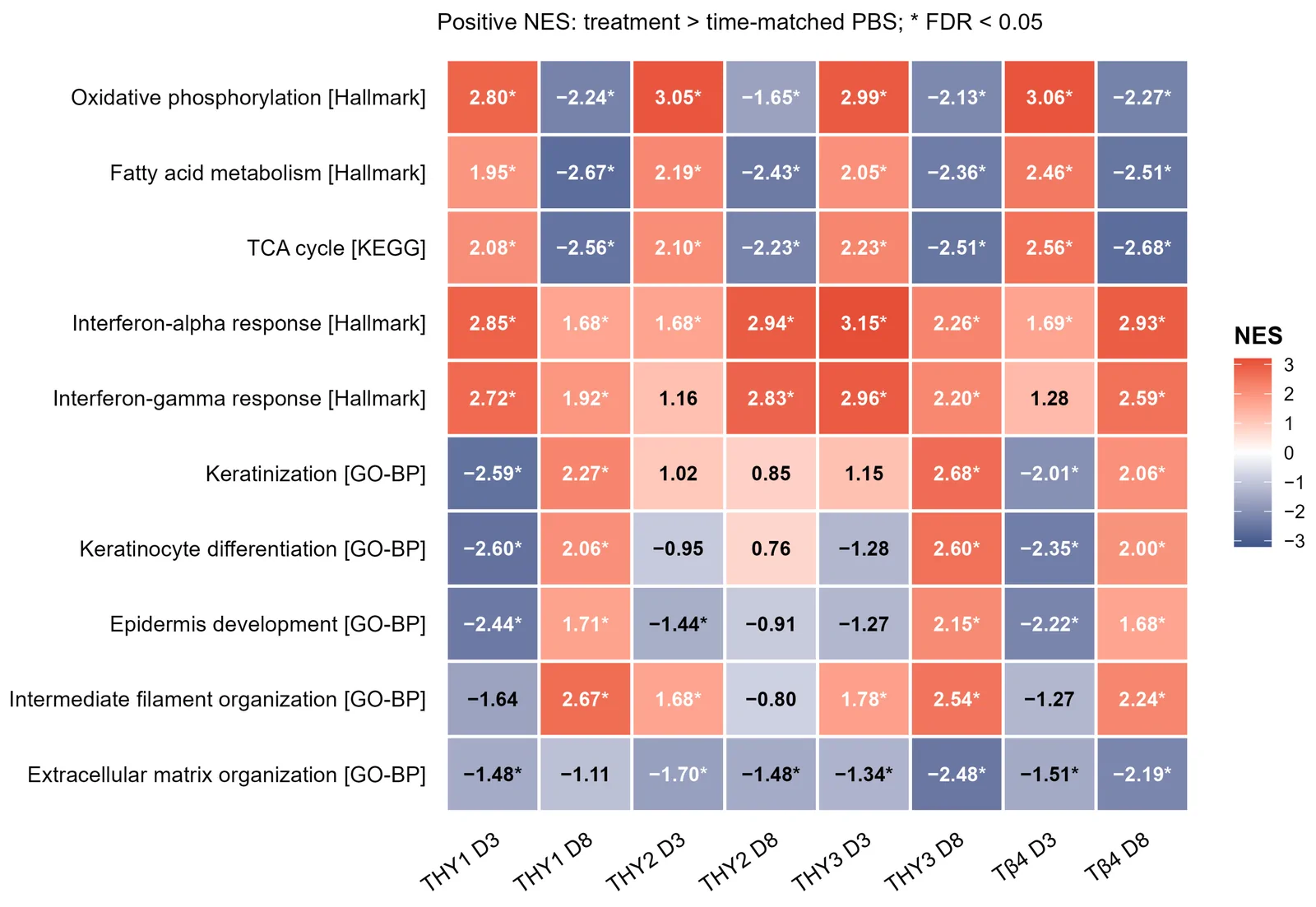
GSEA enrichment scores across treatment groups and time points. Numbers and colors indicate normalized enrichment scores (NES) for the selected gene sets. Positive NES indicates enrichment toward treatment upregulated genes; negative NES indicates enrichment toward PBS upregulated genes. Genes were ranked by the DESeq2 Wald statistic. Asterisks indicate Benjamini–Hochberg FDR < 0.05. D3, day 3; D8, day 8. RNA-seq: *n* = 3 mice per group per time point. Significant results are listed in Table S18.

Preranked GSEA of the eight original treatment-versus-time-matched PBS DESeq2 contrasts identified coordinated pathway-level expression patterns (Figure 5; Tables S18 and S19). On day 3, the THY1 and THY3 contrasts contained three and seven DEGs, respectively, and both showed significant gene-set enrichment. Oxidative phosphorylation was positively enriched in all four day-3 contrasts (NES = 2.80–3.06; FDR ≤ 1.3 × 10^−9^) and negatively enriched in all four day-8 contrasts (NES = −2.27 to −1.65; FDR ≤ 3.7 × 10^−4^). Fatty-acid metabolism and the KEGG citrate cycle (TCA cycle) showed corresponding positive enrichment on day 3 and negative enrichment on day 8. Keratinization was positively enriched on day 8 in the THY1 (NES = 2.27, FDR = 1.2 × 10^−5^), THY3 (NES = 2.68, FDR = 1.5 × 10^−8^), and Tβ4 contrasts (NES = 2.06, FDR = 4.1 × 10^−4^). The interferon-α response gene set was positively enriched in all eight contrasts, and the interferon-γ response gene set was significantly enriched in all four day-8 contrasts. The GO biological process term extracellular matrix organization was negatively enriched in seven of the eight contrasts. In THY2 and THY3 on day 8, higher CVFs coexisted with negative enrichment of the extracellular matrix organization gene set.

### 3.6. PPI Network Analysis

STRING networks were constructed to describe associations among proteins encoded by the DEGs. Cytoscape was used to visualize the networks and rank nodes by degree.

In the THY1 and THY3 day-8 networks, many high-degree genes encoded keratin-associated proteins and keratins (Figures S14 and S15). An interferon-associated group of genes in the THY3 day-8 network included the upregulated genes *Oas1a*, *Oas1b*, *Oas1g*, *Oas2*, *Mx2*, *Ddx60*, *Zbp1*, *Usp18*, *Ifi44*, *Slfn1*, *Slfn8*, *Batf2*, *Nlrc5*, and *Tapbp*.

In the THY2 day-3 network (Figure S16), the network included genes associated with inflammatory and innate immune responses (*Nlrp3*, *Clec4e*, *Il1rn*, *Acod1*, *Ccrl2*, and *Hdc* downregulated; *Cxcl13* and *Cxcl14* upregulated) and genes associated with lipid metabolism (*Cyp4a32*, *Cyp4b1*, *Ces1f*, *Thrsp*, and *Scd1* upregulated; *Pcsk9* and *Cyp2e1* downregulated). In the THY2 day-8 network (Figure S17), high-degree genes were mainly associated with interferon responses (*Irf7*, *Zbp1*, *Ddx60*, *Oas1a*, *Oas1g*, *Oas2*, *Oasl2*, *Ifi44*, *Ifi209*, *Ifi203*, *Gbp3*, *Gbp5*, *Gbp7*, *Gbp8*, *Iigp1*, *Xaf1*, and *Apol9b* upregulated).

## 4. Discussion

In this study, we evaluated the effects of three Pa-THY isoforms on wound healing in ob/ob diabetic mice using macroscopic, histological, and transcriptomic approaches, with Tβ4 as a positive control. Topical Pa-THY treatment improved day-8 wound closure relative to PBS, with accompanying increases in CD31-positive area fractions, whereas higher collagen volume fractions were detected in the THY2 and THY3 groups. No significant differences in day-8 wound closure were detected between the Pa-THY groups and Tβ4. These observations extend the previously reported efficacy of Pa-THYs in nondiabetic wound models to the diabetic context [18]. Transcriptomic profiling identified shared responses and time- and isoform-associated expression patterns that provide biological context for these phenotypic findings.

### 4.1. Epithelial Differentiation

On day 8, both THY1 and THY3 showed increased expression of keratin and keratin-associated protein genes relative to their time-matched PBS controls, together with positive enrichment of keratinization in GSEA. *Foxn1* was upregulated in both day-8 contrasts. *Foxn1* contributes to hair-shaft differentiation and organization, and its expression has been localized to keratinocytes at wound margins and within the advancing epithelial tongue during cutaneous repair [31,32]. These expression patterns suggest an epithelial differentiation-related response in THY1- and THY3-treated wounds. The increased expression of hair keratin and keratin-associated protein genes in the THY1 and THY3 groups on day 8 additionally suggests a response involving appendage-associated epithelium. Lineage-tracing studies have demonstrated that hair-follicle bulge cells contribute progeny to the epidermis after injury, while single-cell analysis has shown that follicular stem-cell progeny acquire an interfollicular epidermal identity during wound repair [33,34]. These hair-associated expression patterns may partly reflect contributions from follicular epithelium in the sampled wound margins. On day 8, *Il1f10* was upregulated in both THY2 and THY3 relative to PBS, connecting these treatment-associated responses with epithelial differentiation and cytokine regulation. *Il1f10* encodes IL-38, whose expression increases during human keratinocyte differentiation; experimental overexpression promotes differentiation-associated features and reduces proliferation [35]. IL-38 also modulates cytokine production in immune-cell systems, with effects that depend on the cellular and inflammatory context [36]. Increased *Il1f10* expression in the day-8 THY2 and THY3 profiles may therefore accompany both epithelial differentiation and changes in epithelial–immune communication during repair.

### 4.2. Immune-Associated Responses

Persistent macrophage inflammasome activity and IL-1β signaling contribute to impaired diabetic wound repair, and experimental inhibition of these pathways improves healing in diabetic mice [37,38]. In the THY2-versus-PBS comparison on day 3, *Nlrp3* was downregulated, whereas *Cxcl13* and *Cxcl14* were upregulated. *Cxcl13* regulates CXCR5-dependent lymphocyte positioning, and *Cxcl14* has demonstrated chemotactic activity toward human monocytes [39,40]. The THY3-versus-PBS comparison on day 3 also showed increased *Slamf8* expression. SLAMF8 negatively regulates macrophage NOX2 activity and thus participates in the control of oxidative responses [41].

Interferon-associated expression was shared more broadly across treatments. The interferon-α response gene set was positively enriched in all eight comparisons, while the interferon-γ response was positively enriched in all four day-8 comparisons. Upregulation of *Oas1a*, *Oas2*, *Ddx60*, and *Zbp1* in both THY2 and THY3 on day 8 further identified an interferon-responsive component of these responses. Interferon signaling participates in tissue repair as well as host defense. Plasmacytoid dendritic cell-derived type I interferons promote re-epithelialization in mouse skin, and microbiota-dependent interferon responses can stimulate growth-factor production by macrophages and fibroblasts during repair [42,43]. The shared enrichment may therefore reflect changes in injury-related communication among immune, epithelial, and stromal compartments. Regulatory genes accompanied these interferon-responsive transcripts. In THY3-treated wounds, *Usp18* was upregulated on day 8. The encoded protein, USP18, negatively regulates type I interferon signaling, making feedback regulation a plausible component of this response [44]. THY2-treated wounds also showed increased *Irf7* expression on day 8. Beyond its role in interferon signaling, IRF7 has been shown to regulate CTSS expression and matrix-related responses in human skin fibroblasts [45]. These findings suggest that the interferon-associated changes may involve both immune signaling and stromal responses during wound repair.

The day-8 THY3 response additionally included increased expression of *Gzma*, *Prf1*, *Nkg7*, and *Tigit*, accompanied by higher NK- and T-cell marker-associated scores. NKG7 supports lytic-granule exocytosis and perforin-dependent cytotoxicity, whereas TIGIT can inhibit NK-cell cytotoxic activity [46,47]. Their increased expression suggests that both effector and inhibitory immune programs contributed to the THY3-associated lymphocyte signature. Such responses can have different consequences for repair: NK-cell depletion improved re-epithelialization and collagen deposition in diabetic mice in a recent experimental study [48]. The THY3 findings may thus reflect changes in lymphocyte-associated activity and cellular representation within repairing tissue, rather than a uniformly pro-reparative immune response.

### 4.3. Metabolic Responses

The early THY2 response included increased expression of *Scd1*, *Thrsp*, and *Ces1f* on day 3, suggesting changes in lipid synthesis and handling. SCD1 catalyzes monounsaturated fatty-acid synthesis, and skin-specific *Scd1* deficiency disrupts lipid and retinol homeostasis and produces inflammatory skin abnormalities [49]. THRSP also promotes lipogenesis in hepatic models [50]. These functional associations suggest that the lipid-related transcriptional response in THY2-treated wounds may be relevant to the maintenance and remodeling of the local lipid environment during early repair. Lipid biosynthesis also supports cellular functions involved in tissue repair. Zuo et al. demonstrated that fatty-acid synthesis sustains growth-factor expression and supplies phospholipids for expansion of the secretory apparatus in reparative macrophages [51]. The concurrent lipid- and immune-associated expression changes in the day-3 THY2 profile may therefore reflect coordination between lipid handling and the biosynthetic and secretory requirements of the early wound response.

Beyond these THY2-associated changes, oxidative phosphorylation, fatty-acid metabolism, and citrate-cycle gene sets were positively enriched in all four treatment-versus-PBS comparisons on day 3 and negatively enriched on day 8. This shared pattern suggests that the metabolic transcriptional response to treatment differed between the two sampling times. Such differences may involve the functional states of cells participating in repair: epidermal basal cells exhibit distinct metabolic preferences that become spatially organized during re-epithelialization [52]. Human diabetic-ulcer profiling has also identified healing-associated differences in fibroblast states and immune-cell composition, which could influence tissue-level expression patterns [53]. Thus, the contrasting enrichment of oxidative phosphorylation, fatty-acid metabolism, and citrate-cycle gene sets may reflect differences in the metabolic states of wound cells at the two sampling times.

### 4.4. Integration of Phenotypic and Transcriptomic Findings

The improved wound closure after Pa-THY treatment was accompanied by shared and group-associated transcriptional responses involving epithelial differentiation, immune signaling, and metabolism. In THY1 and THY3, the day-8 epithelial signatures are consistent with the more complete epithelial coverage observed histologically. The early inflammatory- and lipid-related expression changes in THY2 suggest that alterations in the local wound environment may also accompany repair. These patterns raise the possibility that the isoforms influence partly overlapping aspects of the tissue response, with epithelial differentiation being more prominently in some treatment-versus-PBS profiles. The shared interferon and metabolic enrichment patterns further suggest that these group-associated features occur within broader responses to treatment.

Histological findings provide additional context for these transcriptional patterns. Higher CD31-positive area fractions may reflect vascular-associated tissue changes, while the higher CVF in THY2 and THY3 indicates a greater proportion of collagen-positive tissue in the analyzed sections. In these two groups, higher day-8 CVFs coexisted with negative enrichment of the extracellular matrix organization gene set. Collagen staining reflects matrix present at collection, whereas this broad gene set captures ongoing matrix-related transcription. Differences in their direction may therefore involve the timing of matrix deposition and turnover, as well as the cellular composition of the sampled tissue. Partial correspondence and temporal offsets between transcript and protein changes have been reported during human wound healing, while single-cell studies have identified substantial variation in fibroblast states and immune-cell composition in diabetic ulcers [53,54]. These temporal and cellular differences may help explain how improved closure and greater collagen-positive areas coexist with distinct matrix-related transcriptional responses.

## 5. Limitations

This study has several limitations. The histological and RNA-seq cohorts were small (*n* = 3 mice per group per time point), limiting statistical power and the precision of effect estimates. Only male ob/ob mice were studied, restricting generalizability across sexes and diabetes models. Blood glucose and body weight were not monitored longitudinally, and diet composition could not be verified, limiting assessment of metabolic and nutritional factors that may influence repair. Residual endotoxin was not quantified; contributions of preparation-related contaminants to the immune- and interferon-associated responses therefore cannot be excluded. Finally, the transcriptional findings lacked independent molecular and functional validation, leaving the contributions of the identified pathways to improved closure unresolved. Future studies should combine rigorously characterized, endotoxin-quantified protein preparations with larger cohorts, longitudinal sampling, cell-resolved profiling, and targeted protein and functional validation to determine which responses contribute to repair and whether the benefits extend to sustained tissue function.

## 6. Conclusions

Topical treatment with THY1, THY2, or THY3 improved wound closure on day 8 in male ob/ob mice, accompanied by higher CD31-positive area fractions. The THY2 and THY3 groups also showed higher collagen volume fractions, consistent with greater local collagen accumulation during repair. No statistically significant differences in day-8 wound closure were detected between the Pa-THY groups and Tβ4. Transcriptomic analyses revealed shared and isoform-associated responses involving epithelial differentiation, immune signaling, and metabolism. GSEA identified positive enrichment of metabolic gene sets on day 3 and negative enrichment on day 8, together with shared interferon-associated enrichment and day-8 keratinization enrichment in the THY1, THY3, and Tβ4 groups. Together, these findings demonstrate improved wound closure accompanied by tissue-level and transcriptional changes, and they support further preclinical evaluation of recombinant *P. americana* thymosin isoforms for diabetic wound repair.

## Data Availability

The raw RNA-seq data are available in CNGBdb under accession number CNP0003984 (https://db.cngb.org/data_resources/project/CNP0003984) (accessed on 4 February 2023).

## Supplementary Materials

The following supporting information can be downloaded at: https://www.mdpi.com/article/10.3390/genes17101296/s1, Table S1: Wound-closure fractions in each group (0–1); Table S2: Pairwise comparisons of wound-closure percentages between groups at each time point; Table S3: RNA-seq sample information, mapping rates, and sequencing QC metrics; Table S4: Numbers of DEGs; Table S5: GO and KEGG enrichment results for upregulated DEGs in THY1 VS PBS (day 8);Table S6: GO and KEGG enrichment results for downregulated DEGs in THY2 VS PBS (day 3); Table S7: GO and KEGG enrichment results for upregulated DEGs in THY2 VS PBS (day 3); Table S8: GO and KEGG enrichment results for upregulated DEGs in THY2 VS PBS (day 8); Table S9: GO and KEGG enrichment results for upregulated DEGs in THY3 VS PBS (day 3); Table S10: GO and KEGG enrichment results for upregulated DEGs in THY3 VS PBS (day 8); Table S11: GO and KEGG enrichment results for downregulated DEGs in THY3 VS PBS (day 8); Table S12: GO and KEGG enrichment results for downregulated DEGs in Tβ4 VS PBS (day 3); Table S13: GO and KEGG enrichment results for upregulated DEGs in Tβ4 VS PBS (day 3); Table S14: GO and KEGG enrichment results for downregulated DEGs in Tβ4 VS PBS (day 8); Table S15: GO and KEGG enrichment results for upregulated DEGs in Tβ4 VS PBS (day 8).; Table S16: Cell-type-associated marker expression scores derived from bulk RNA-seq data; Table S17: Marker gene sets used to calculate cell-type-associated expression scores; Table S18: Significant preranked GSEA results from the eight original treatment-versus-time-matched PBS DESeq2 comparisons; Table S19: Leading-edge genes for the pathways displayed in Figure 5 across the eight original pairwise DESeq2 comparisons.; Figure S1: CD31-positive area fraction in each group on days 3, 5, and 8 after wounding; Figure S2: Collagen volume fraction in each group on days 3, 5, and 8 after wounding; Figure S3: H&E staining of wound tissue on days 3 and 8 after wounding; Figure S4: Agarose-gel electrophoresis of total RNA from wound tissue, showing 28S, 18S, and 5S rRNA bands; Figure S5: Hierarchical clustering heatmaps of row-wise Z-scaled DEG expression in each treatment-versus-time-matched PBS comparison; Figure S6: GO over-representation analysis of DEGs in the Tβ4-versus-time-matched PBS contrasts; Figure S7: Heatmap of Z-scaled cell-type-associated marker expression scores across treatment groups and time points; Figure S8: Myeloid-cell-associated marker expression scores on days 3 and 8; Figure S9: Lymphoid-cell-associated marker expression scores on days 3 and 8; Figure S10: Stromal-cell-associated marker expression scores on days 3 and 8; Figure S11: Preranked GSEA enrichment plots for oxidative phosphorylation across the eight treatment-versus-time-matched PBS contrasts; Figure S12: Preranked GSEA enrichment plots for keratinization on day 8; Figure S13: Preranked GSEA enrichment plots for interferon-α and interferon-γ responses; Figure S14: PPI network derived from DEGs in the THY1-versus-time-matched PBS comparison on day 8; Figure S15: PPI network derived from DEGs in the THY3-versus-time-matched PBS comparison on day 8; Figure S16: PPI network derived from DEGs in the THY2-versus-time-matched PBS comparison on day 3; Figure S17: PPI network derived from DEGs in the THY2-versus-time-matched PBS comparison on day 8. Figure S18: Mean–dispersion relationships for the eight original treatment-versus-time-matched PBS DESeq2 models.

## Abbreviations

The following abbreviations are used in this manuscript:

AGE	Agarose gel electrophoresis
BAM	Binary alignment/map
BCA	Bicinchoninic acid
CD8	Cluster of differentiation 8
CD31	Cluster of differentiation 31
CNGBdb	China National GeneBank DataBase
CPM	Counts per million
CVF	Collagen volume fraction
DEG	Differentially expressed gene
DFU	Diabetic foot ulcer
FDR	False discovery rate
GO	Gene ontology
GO-BP	Gene ontology biological process
GSEA	Gene set enrichment analysis
H&E	Hematoxylin and eosin
IPTG	Isopropyl β-D-1-thiogalactopyranoside
ISG	Interferon-stimulated gene
JAK–STAT	Janus kinase–signal transducer and activator of transcription
KEGG	Kyoto Encyclopedia of Genes and Genomes
KRT	Keratin
KRTAP	Keratin-associated protein
LB	Lysogeny broth
log_2_FC	Base-2 logarithm of fold change
mMCP-counter	Murine microenvironment cell populations counter
MSigDB	Molecular Signatures Database
NADPH	Nicotinamide adenine dinucleotide phosphate
NES	Normalized enrichment score
NGS	Next-generation sequencing
NK	Natural killer
NOD	Nucleotide-binding oligomerization domain
OD_600_	Optical density at 600 nm
Pa-THY1–3	Recombinant *P. americana* thymosin isoforms 1–3
Pa-THYs	Recombinant *P. americana* thymosin isoforms
PBS	Phosphate-buffered saline
PCA	Principal component analysis
PPI	Protein–protein interaction
qPCR	Quantitative polymerase chain reaction
QC	Quality control
RNA	Ribonucleic acid
RNA-seq	RNA sequencing
rRNA	Ribosomal RNA
SD	Standard deviation
SDS–PAGE	Sodium dodecyl sulfate–polyacrylamide gel electrophoresis
SPF	Specific pathogen-free
Tβ4	Thymosin β4
TCA	Tricarboxylic acid
TCM	Traditional Chinese medicine
TMM	Trimmed mean of M values
