# Supplementary material for: Comparative Transcriptomic Profiling of Diabetic Wound Responses to Recombinant Periplaneta americana Thymosin Isoforms in Male ob/ob Mice
Source: Genes (Basel). 2026 Oct 16;17(10):1296. doi: 10.3390/genes17101296 (PMC13704345; doi:10.3390/genes17101296)
Supplement: Supplementary file 1 [file genes-17-01296-s001.zip › Additional_file_1_Supplementary_Figures.pdf]

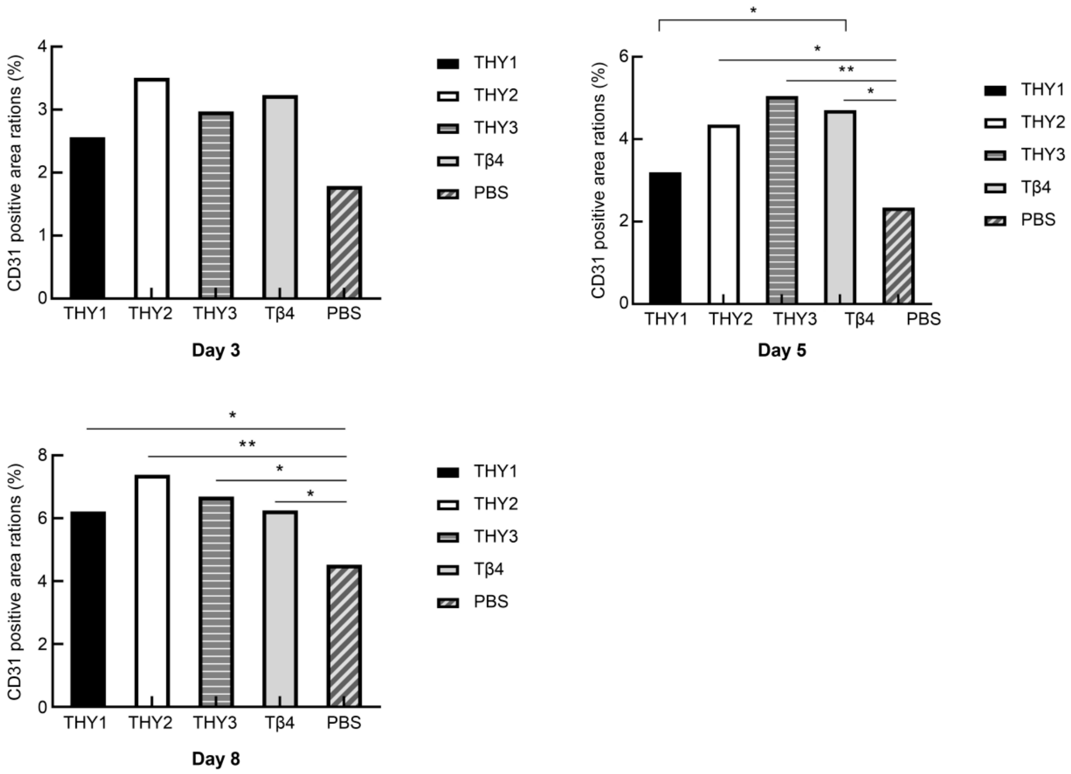

Figure S1. CD31-positive area fraction in each group on days 3, 5, and 8 after wounding. Bars show group means (n = 3 mice per group per time point). Five fields were averaged to obtain one value per mouse. *P < 0.05 and **P < 0.01 versus PBS; #P < 0.05 versus Tβ4.

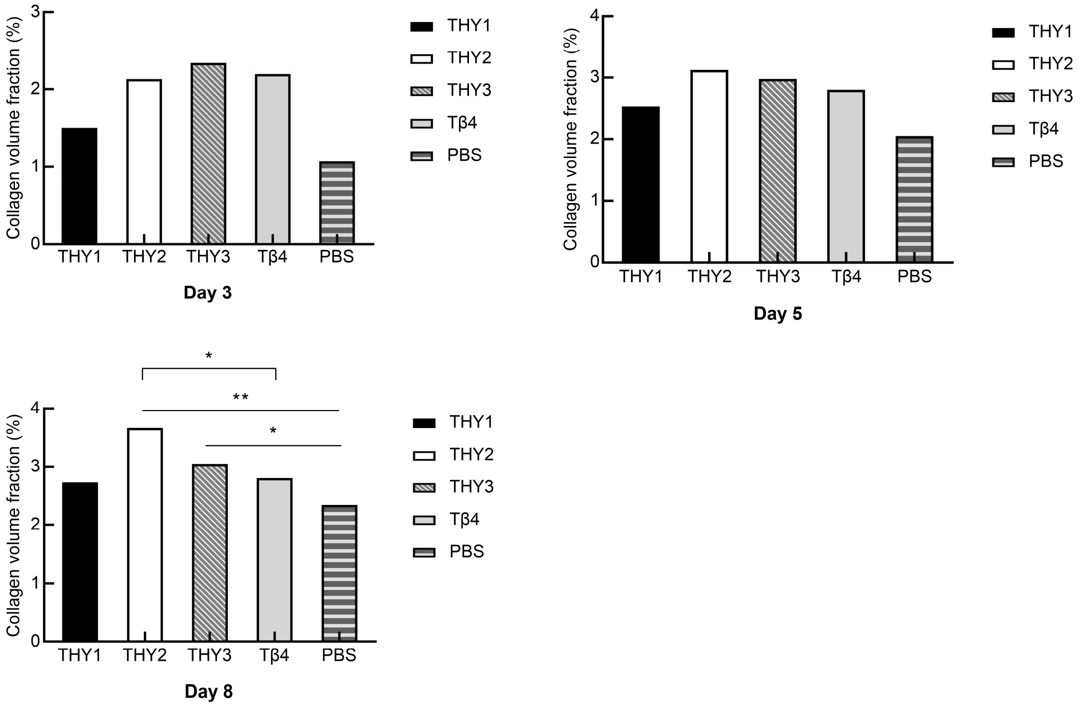

Figure S2. Collagen volume fraction in each group on days 3, 5, and 8 after wounding. Bars show group means (n = 3 mice per group per time point). Five fields were averaged to obtain one value per mouse. *P < 0.05 and **P < 0.01 versus PBS.

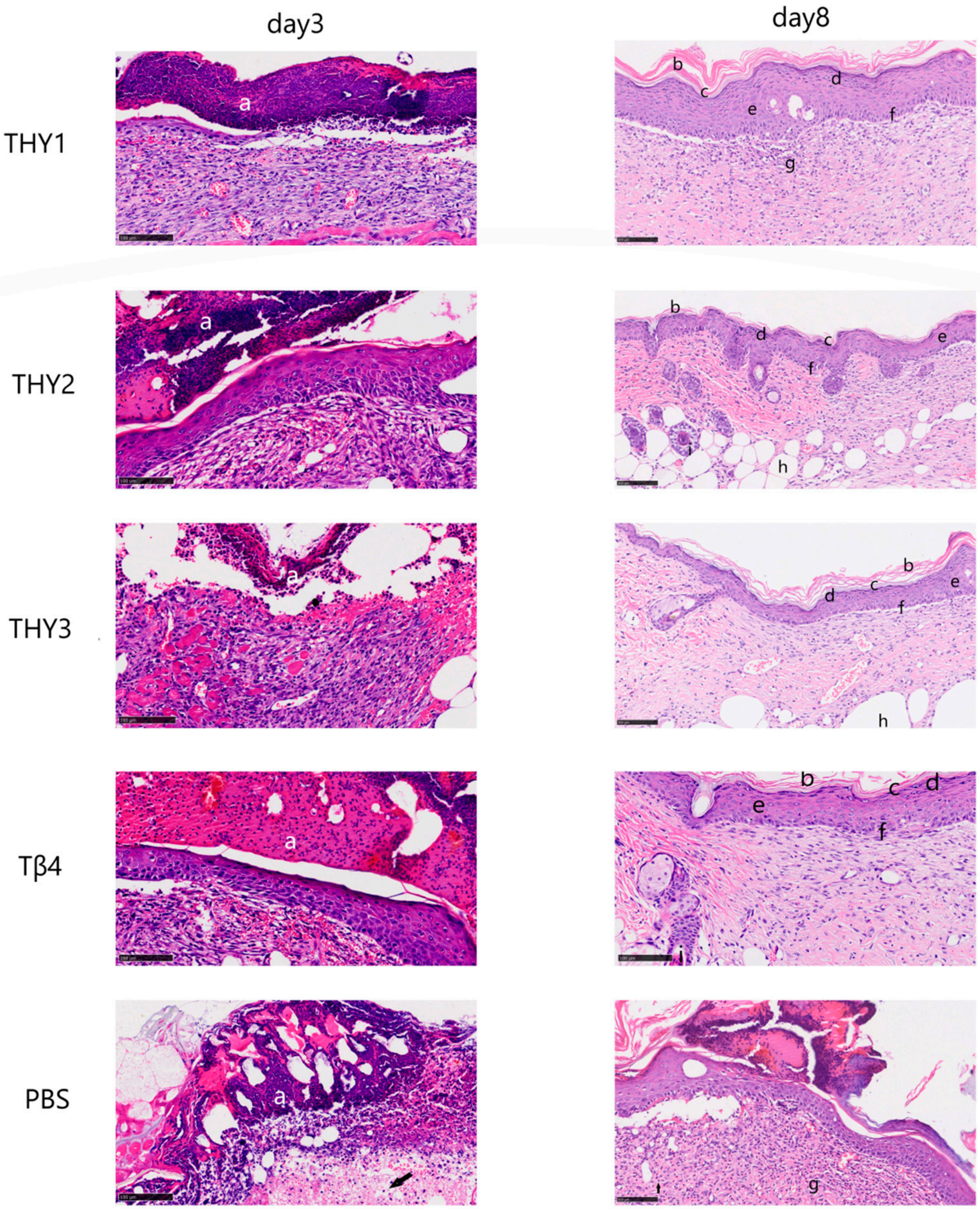

Figure S3. Representative H&E-stained sections of wound tissue on days 3 and 8 after wounding (×200; scale bar, 100 μm). Histological assessment included n = 3 mice per group per time point. a, surface crust and necrotic tissue; b, stratum corneum; c–f, epidermal layers; g, granulation tissue; h, adipose tissue; i, hair follicle; arrowhead, inflammatory-cell-rich area.

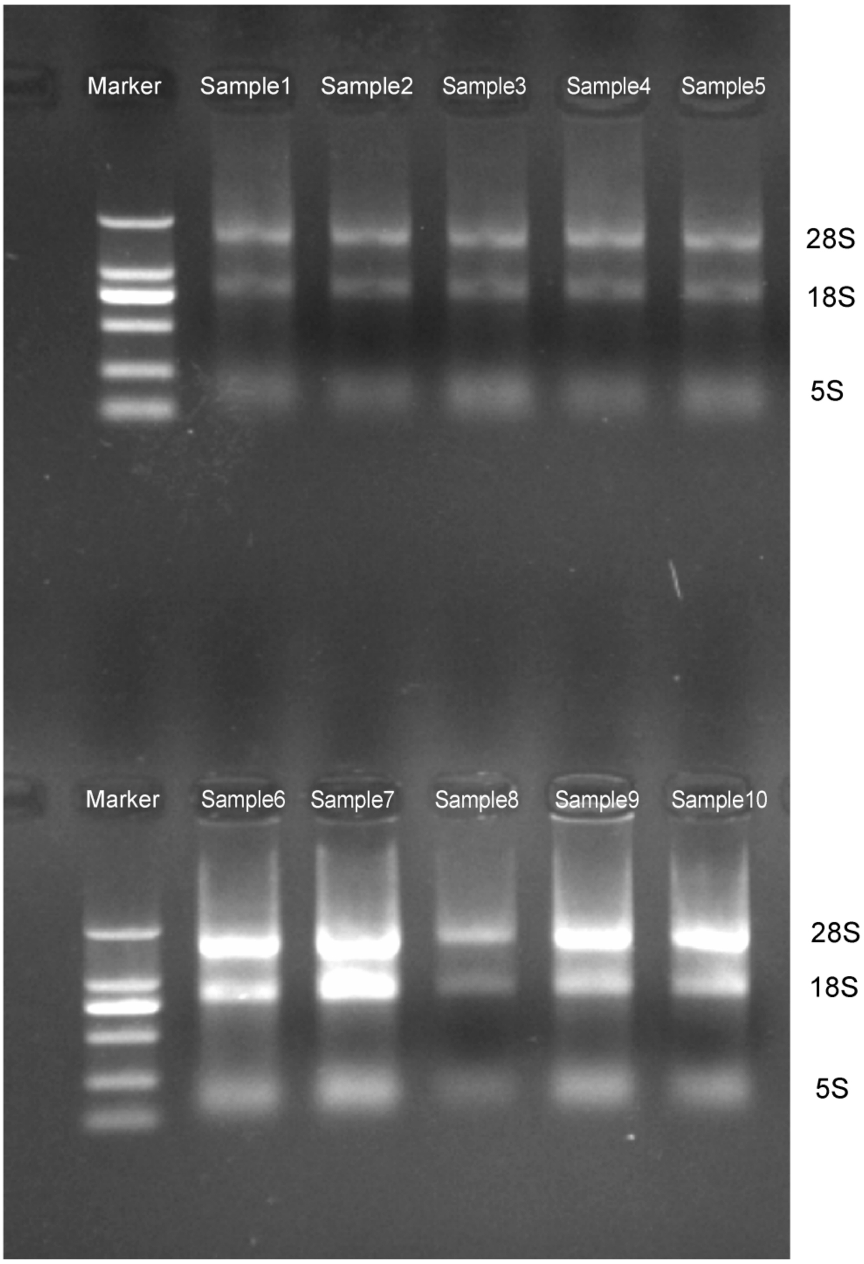

Figure S4. Agarose-gel electrophoresis of total RNA from wound tissue, showing 28S, 18S, and 5S rRNA bands. Ten samples were randomly selected from the RNA-seq cohort of 30 independent mice (n = 3 mice per group per time point).

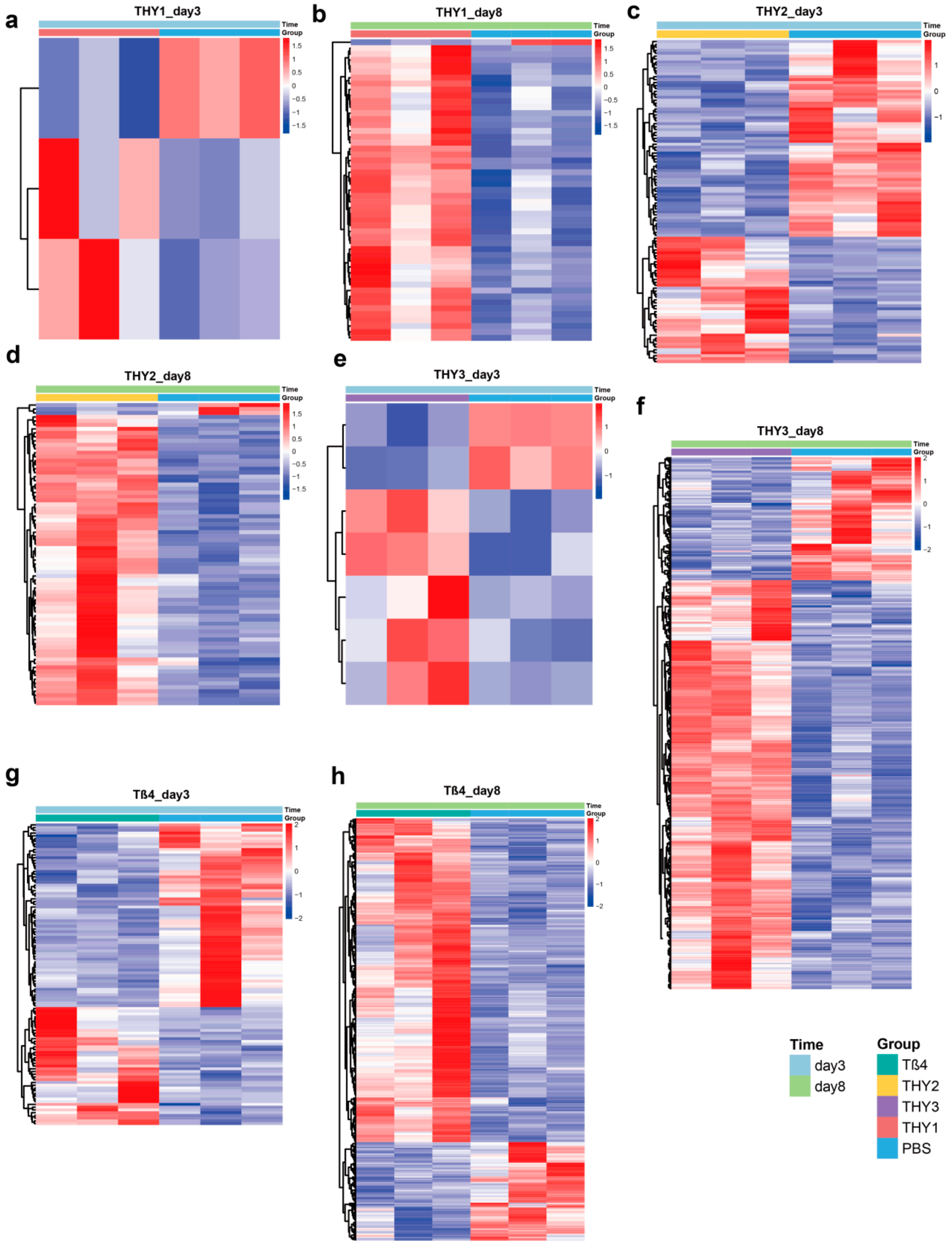

Figure S5. Hierarchical clustering heatmaps of row-wise Z-scaled DEG expression in each treatment-versus-time-matched-PBS comparison (n = 3 mice per group per time point). Colors indicate relative expression within each gene.

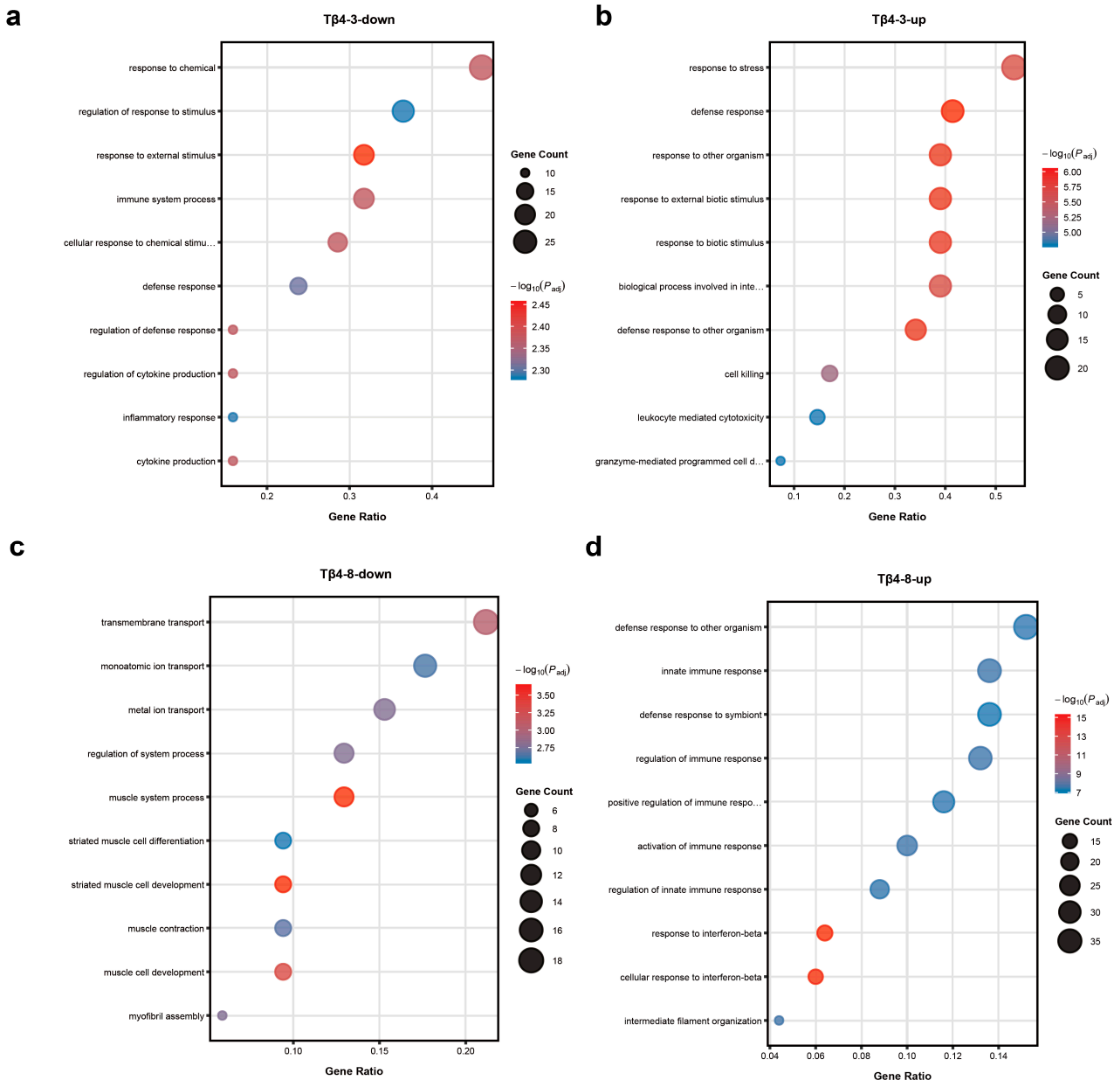

Figure S6. Gene Ontology over-representation analysis of DEGs. Circle size indicates gene count, and color indicates −log10(adjusted P). (a) Downregulated and (b) upregulated genes under Tβ4 on day 3; (c) downregulated and (d) upregulated genes under Tβ4 on day 8. RNA-seq: n = 3 mice per group per time point.

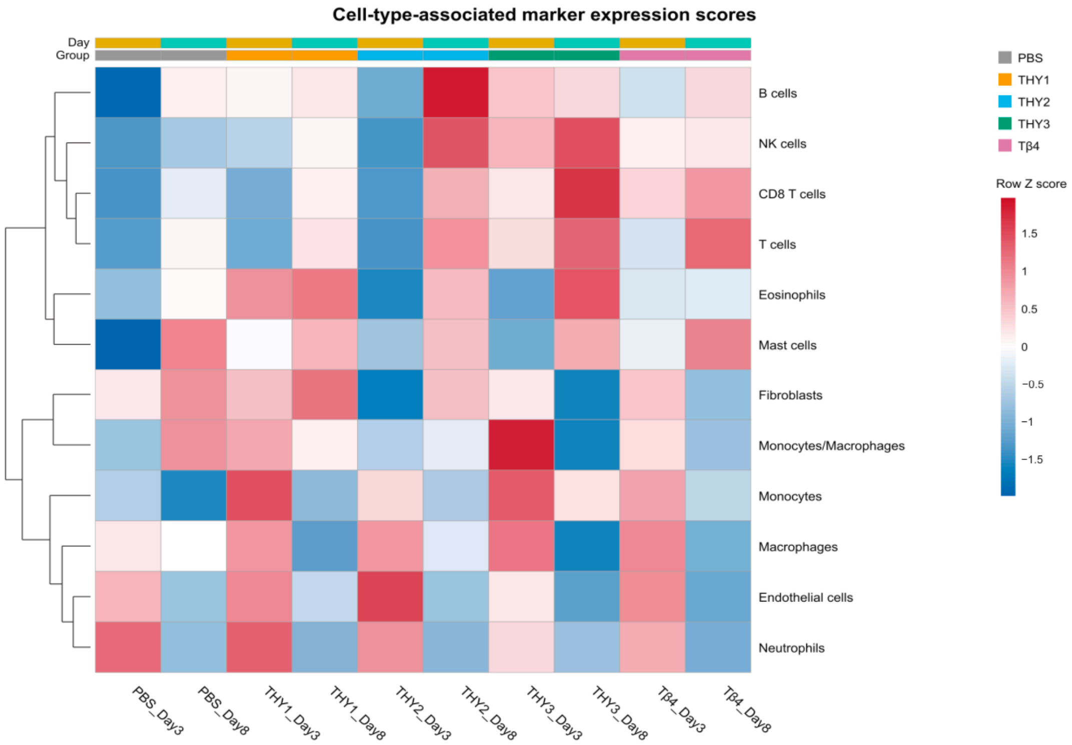

Figure S7. Heatmap of row-wise Z-scaled cell-type-associated marker expression scores across treatment groups and time points. Each column represents the mean of three independent mice in one treatment-by-time group, and each row represents a marker gene set. Z scores were calculated across the ten group means within each marker set. Scores are expression signatures rather than direct cell counts.

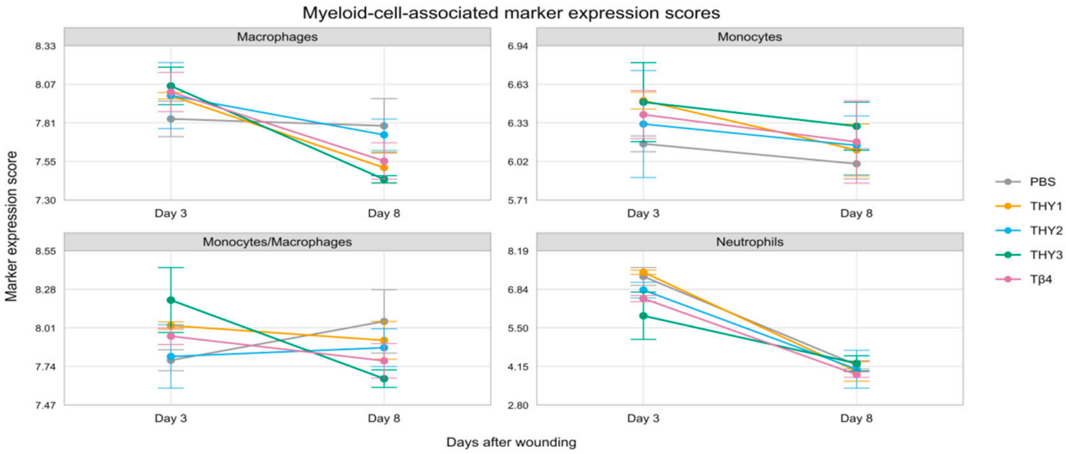

Figure S8. Myeloid-cell-associated marker expression scores on days 3 and 8 after wounding. Scores were calculated as the mean log2(CPM + 1) across detected marker genes. Points and error bars show group means ± SEM (n = 3 independent mice per group per time point). Lines connect group means from different mice at the two time points. Hedges' g and adjusted P values for treatment-versus-PBS comparisons are provided in Table S16. Scores are exploratory expression signatures rather than direct cell counts.

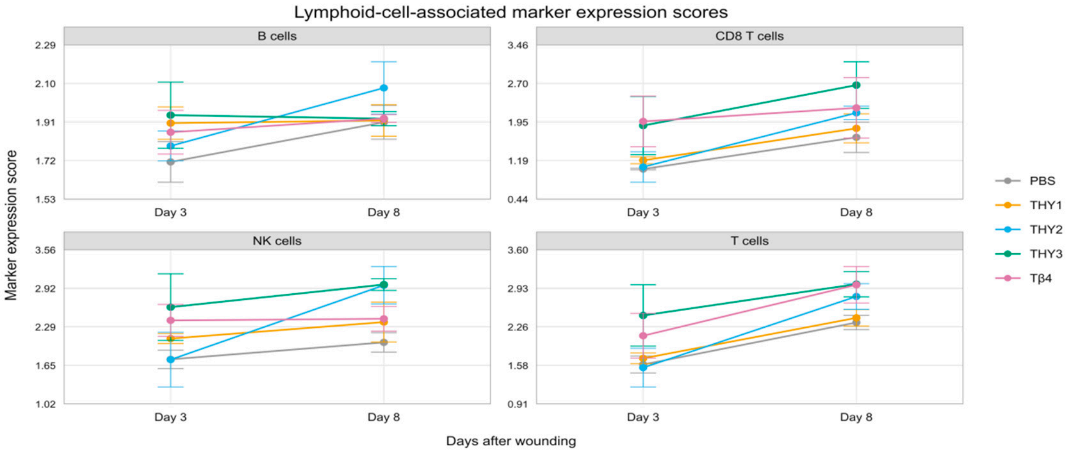

Figure S9. Lymphoid-cell-associated marker expression scores on days 3 and 8 after wounding. Scores were calculated as the mean log2(CPM + 1) across detected marker genes. Points and error bars show group means ± SEM (n = 3 independent mice per group per time point). Lines connect group means from different mice at the two time points. Hedges' g and adjusted P values for treatment-versus-PBS comparisons are provided in Table S16. Scores are exploratory expression signatures rather than direct cell counts.

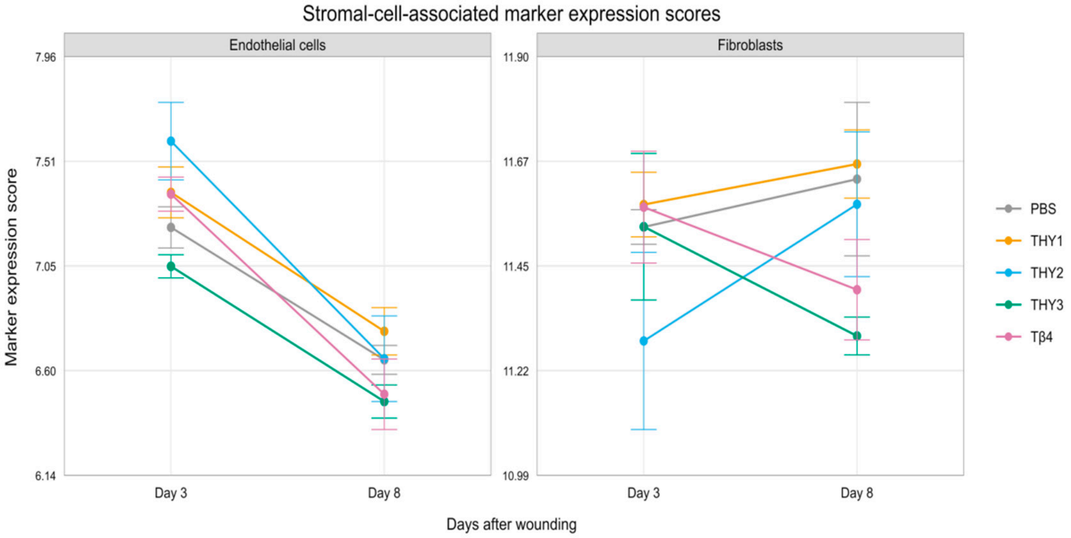

Figure S10. Stromal-cell-associated marker expression scores on days 3 and 8 after wounding. Scores were calculated as the mean log2(CPM + 1) across detected marker genes. Points and error bars show group means ± SEM (n = 3 independent mice per group per time point). Lines connect group means from different mice at the two time points. Hedges' g and adjusted P values for treatment-versus-PBS comparisons are provided in Table S16. Scores are exploratory expression signatures rather than direct cell counts.

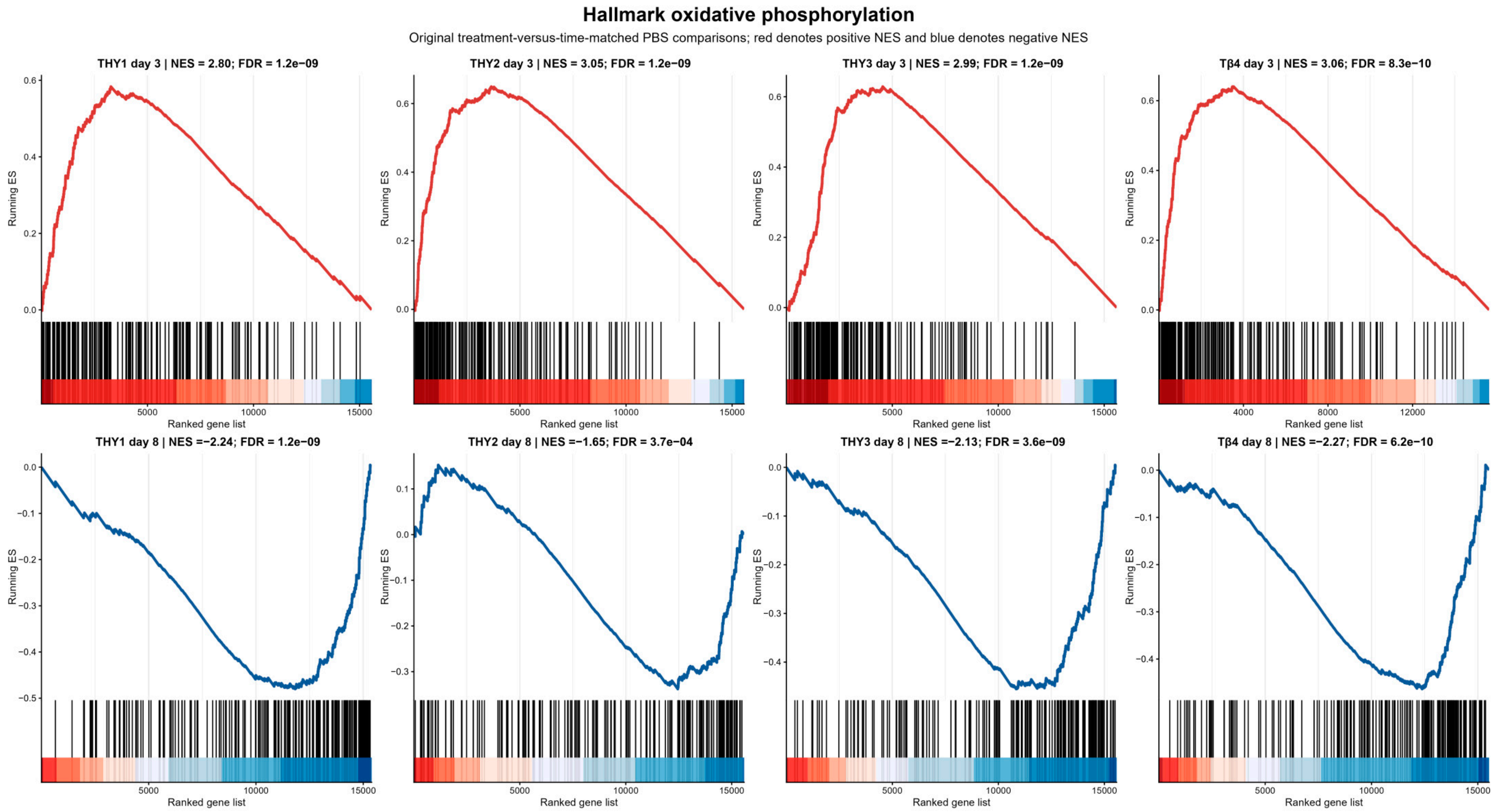

Figure S11. Preranked GSEA enrichment plots for Hallmark oxidative phosphorylation across the eight original treatment-versus-time-matched-PBS comparisons. Genes were ranked by the DESeq2 Wald statistic. Positive normalized enrichment scores (NESs) on day 3 and negative NESs on day 8 indicate opposite treatment-associated enrichment directions relative to the corresponding time-matched PBS group. Day-3 and day-8 contrasts were analyzed separately and do not constitute a formal treatment-by-time interaction. RNA-seq: n = 3 mice per group per time point.

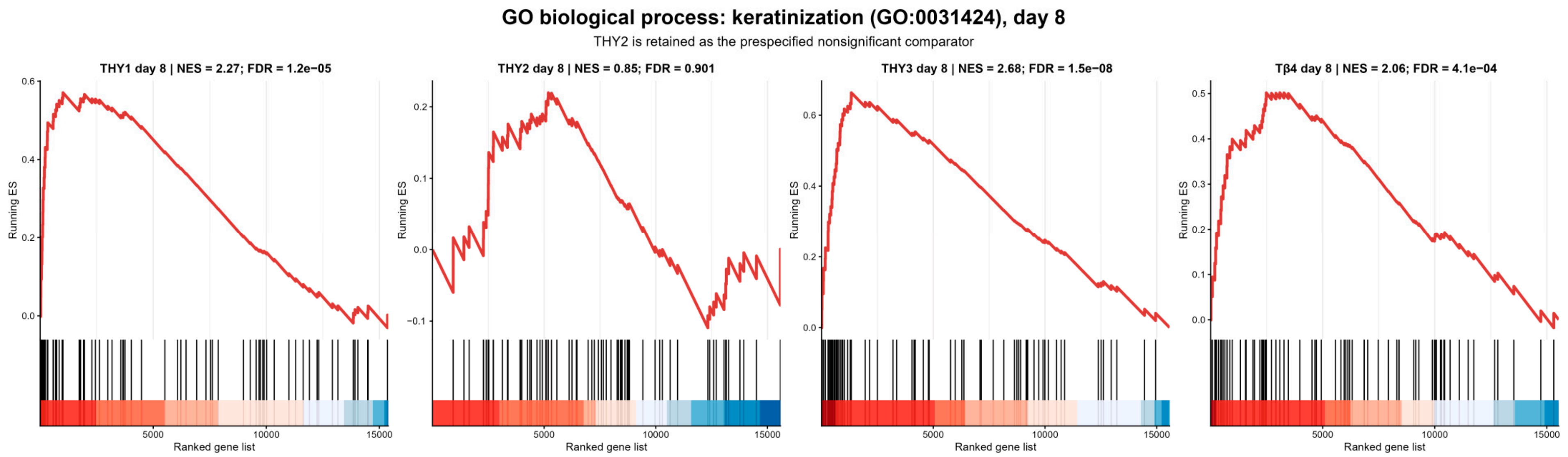

Figure S12. Preranked GSEA enrichment plots for GO keratinization (GO:0031424) on day 8. Keratinization was positively enriched under THY1, THY3, and Tβ4, but was not significant under THY2. Genes were ranked by the DESeq2 Wald statistic for each treatment-versus-time-matched-PBS comparison; NES and Benjamini–Hochberg FDR are shown in each panel. RNA-seq: n = 3 mice per group per time point.

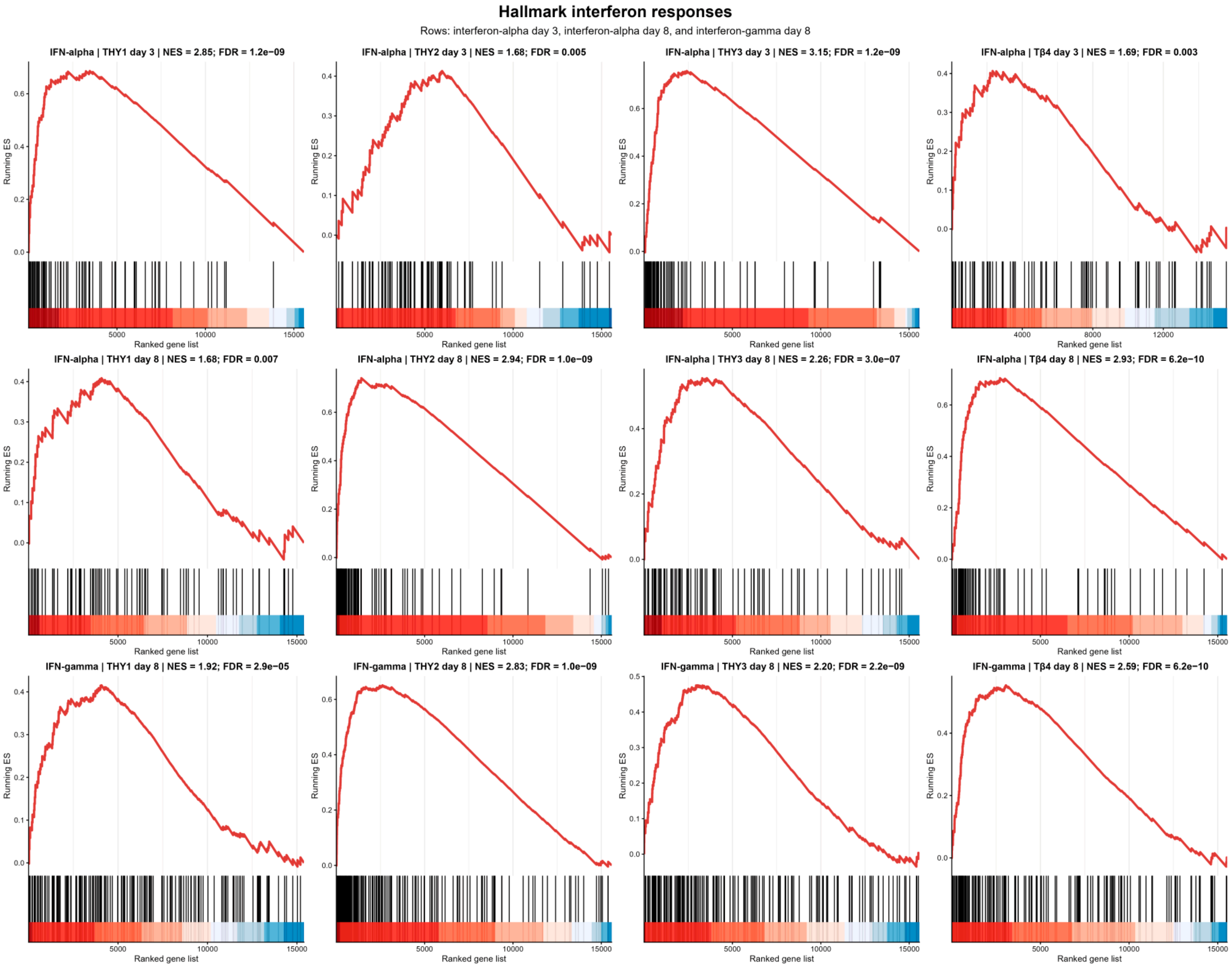

Figure S13. Preranked GSEA enrichment plots for Hallmark interferon-alpha and interferon-gamma responses. Interferon-alpha response was positively enriched in all eight treatment-versus-time-matched-PBS comparisons, whereas interferon-gamma response was significantly enriched in all four day-8 comparisons. Genes were ranked by the DESeq2 Wald statistic; NES and Benjamini–Hochberg FDR are shown in each panel. RNA-seq: n = 3 mice per group per time point.

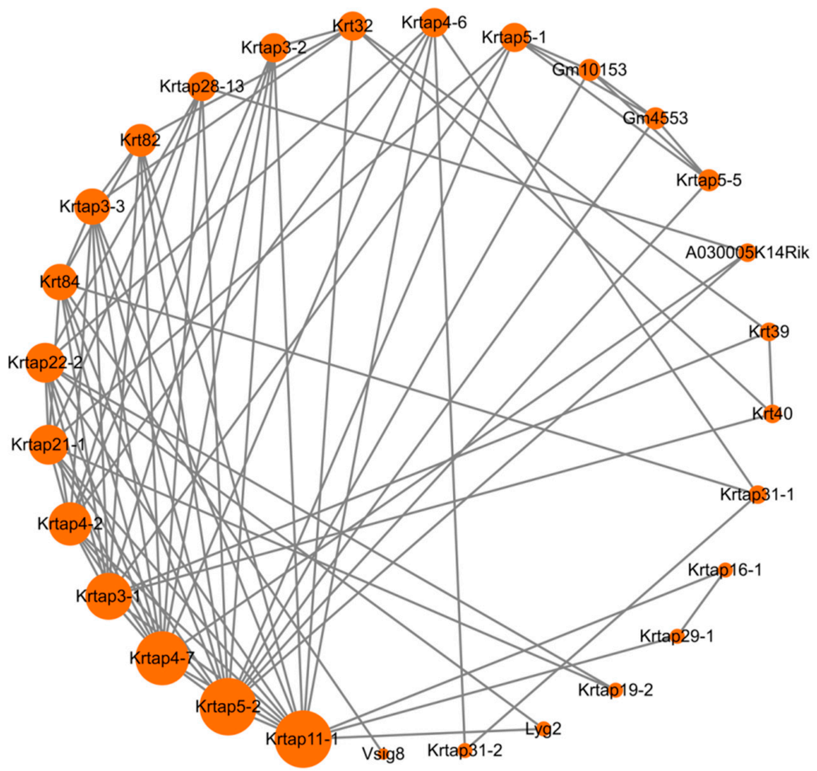

Figure S14. Protein-protein interaction (PPI) network derived from DEGs in the THY1-versus-time-matched-PBS comparison on day 8. Nodes represent DEG-encoded proteins, labelled by their corresponding gene symbols; edges represent STRING associations. Node size is proportional to node degree, highlighting relatively highly connected proteins. RNA-seq: n = 3 mice per group per time point.

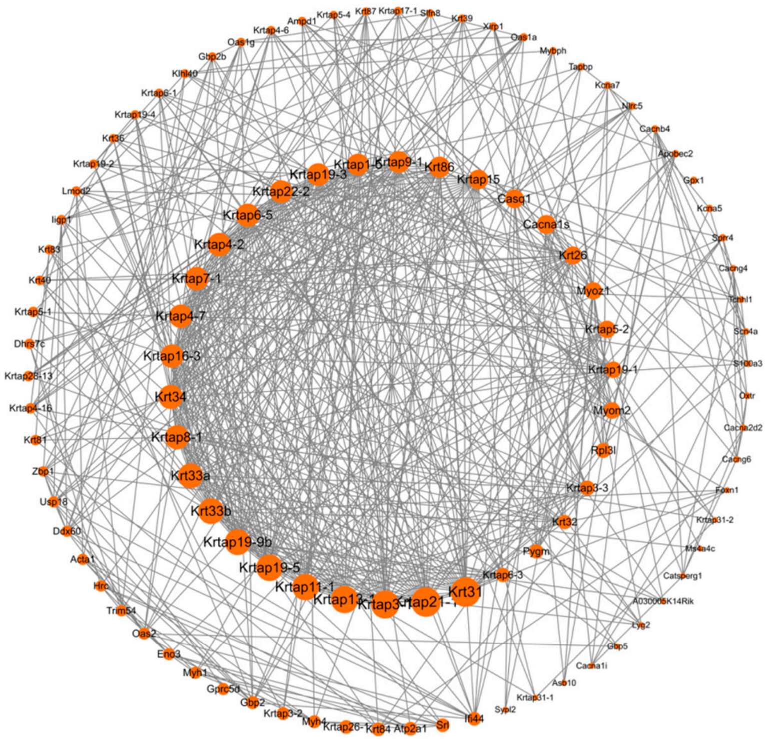

Figure S15. Protein-protein interaction (PPI) network derived from DEGs in the THY3-versus-time-matched-PBS comparison on day 8. Nodes represent DEG-encoded proteins, labelled by their corresponding gene symbols; edges represent STRING associations. Node size is proportional to node degree, highlighting relatively highly connected proteins. RNA-seq: n = 3 mice per group per time point.

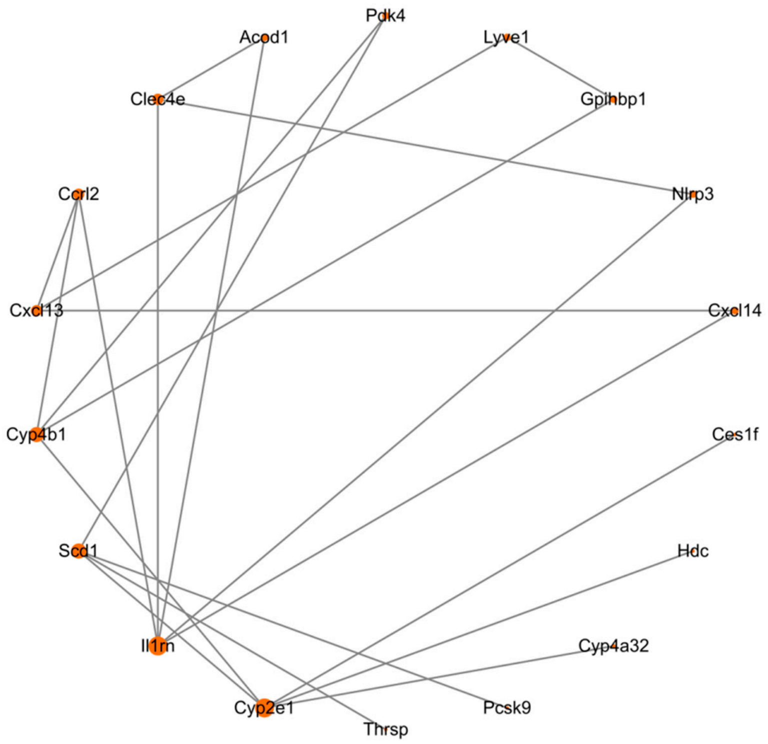

Figure S16. Protein-protein interaction (PPI) network derived from DEGs in the THY2-versus-time-matched-PBS comparison on day 3. Nodes represent DEG-encoded proteins, labelled by their corresponding gene symbols; edges represent STRING associations. Node size is proportional to node degree, highlighting relatively highly connected proteins. RNA-seq: n = 3 mice per group per time point.

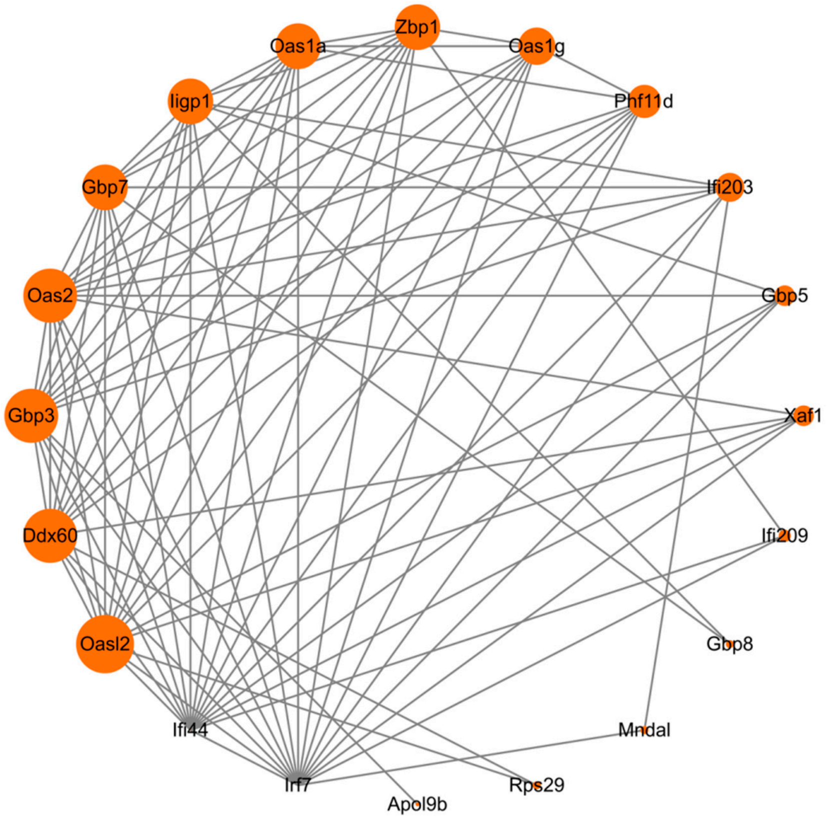

Figure S17. Protein-protein interaction (PPI) network derived from DEGs in the THY2-versus-time-matched-PBS comparison on day 8. Nodes represent DEG-encoded proteins, labelled by their corresponding gene symbols; edges represent STRING associations. Node size is proportional to node degree, highlighting relatively highly connected proteins. RNA-seq: n = 3 mice per group per time point.

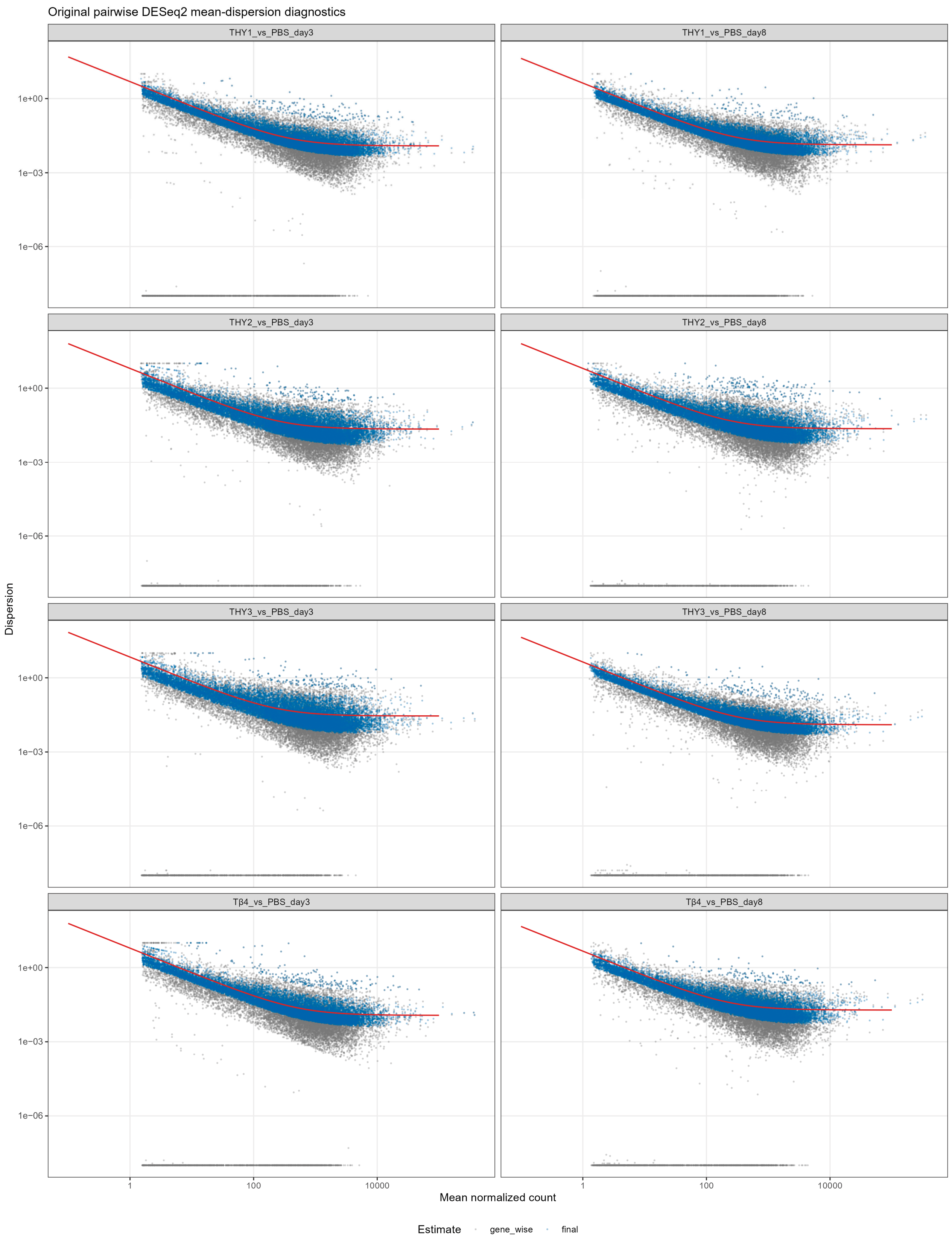

Figure S18. Mean–dispersion relationships for the eight original treatment-versus-time-matched-PBS DESeq2 models. Columns show days 3 and 8, and rows show THY1, THY2, THY3, and Tβ4. Gray points represent gene-wise dispersion estimates, blue points represent final estimates, and red curves indicate fitted dispersion trends. Each model included three treatment and three time-matched PBS samples and retained genes with a total count of at least 10 across the six samples. Models were reconstructed from the original count matrix using DESeq2 version 1.52.0 with condition as the design factor. Both axes are logarithmic. No samples were excluded or count values replaced.
